# Advanced injectable hydrogels for cartilage tissue engineering

**DOI:** 10.3389/fbioe.2022.954501

**Published:** 2022-09-08

**Authors:** Senbo Zhu, Yong Li, Zeju He, Lichen Ji, Wei Zhang, Yu Tong, Junchao Luo, Dongsheng Yu, Qiong Zhang, Qing Bi

**Affiliations:** ^1^ Center for Rehabilitation Medicine, Department of Orthopedics, Zhejiang Provincial People’s Hospital (Affiliated People’s Hospital, Hangzhou Medical College), Hangzhou, China; ^2^ Department of Orthopedics, The Second Affiliated Hospital and Yuying Children’s Hospital of Wenzhou Medical University, Wenzhou, China; ^3^ Zhejiang University of Technology, Hangzhou, China; ^4^ Center for Operating Room, Department of Nursing, Zhejiang Provincial People’s Hospital (Affiliated People’s Hospital, Hangzhou Medical College), Hangzhou, China

**Keywords:** injectable hydrogels, tissue engineering, cartilage defect, osteoarthritis, advanced

## Abstract

The rapid development of tissue engineering makes it an effective strategy for repairing cartilage defects. The significant advantages of injectable hydrogels for cartilage injury include the properties of natural extracellular matrix (ECM), good biocompatibility, and strong plasticity to adapt to irregular cartilage defect surfaces. These inherent properties make injectable hydrogels a promising tool for cartilage tissue engineering. This paper reviews the research progress on advanced injectable hydrogels. The cross-linking method and structure of injectable hydrogels are thoroughly discussed. Furthermore, polymers, cells, and stimulators commonly used in the preparation of injectable hydrogels are thoroughly reviewed. Finally, we summarize the research progress of the latest advanced hydrogels for cartilage repair and the future challenges for injectable hydrogels.

## 1 Introduction

Osteoarthritis (OA) is the most common chronic disease of joints, affecting approximately 90 million adults in the United States alone (approximately 37% of the adult population) and hundreds of millions worldwide (Krishnan and Grodzinsky, 2018; Luo et al., 2021). It is characterized by degeneration and defect of articular cartilage, which can cause joint pain, reduced mobility, and stiffness (Das and Farooqi, 2008). Unlike most other organizations, cartilage is a type of special connective tissue without blood vessels, nerves, and lymph nodes, characterized by immersing chondrocytes in ECM, which consists mainly of a matrix (polysaccharides), fibrous components (fibrin), and interstitial fluid (mainly water) (Armiento et al., 2019; Sirong et al., 2020). Therefore, cartilage cannot repair itself due to insufficient nutritional support and proper progenitor cell differentiation. When cartilage defects go untreated, joints inevitably deteriorate, leading to OA and disability (Simon and Jackson, 2006; Gao et al., 2014).

Non-surgical conservative treatment and drug (painkillers and NSAIDs) therapy can effectively relieve pain in the early stages of articular cartilage lesion but cannot reverse cartilage degeneration defect (Poddar and Widstrom, 2017). Transplantation (using allogeneic or autologous cells or tissues) and stimulation (stimulating self-repair of articular cartilage) are commonly used in the late treatment of OA (Tuan et al., 2013). The former includes allograft or autologous cartilage transplantation, perichondrium and periosteum transplantation, osteochondral transplantation, ACI, and other graft repair techniques. The latter include joint cleansing and debridement, cartilage grinding and shaping, microfractures, drilling, osteotomy, and joint traction (Fuggle et al., 2020). For patients with severe OA, severe invasive total joint replacement surgery is generally considered the last resort (Katz et al., 2021).

**FIGURE 1:**
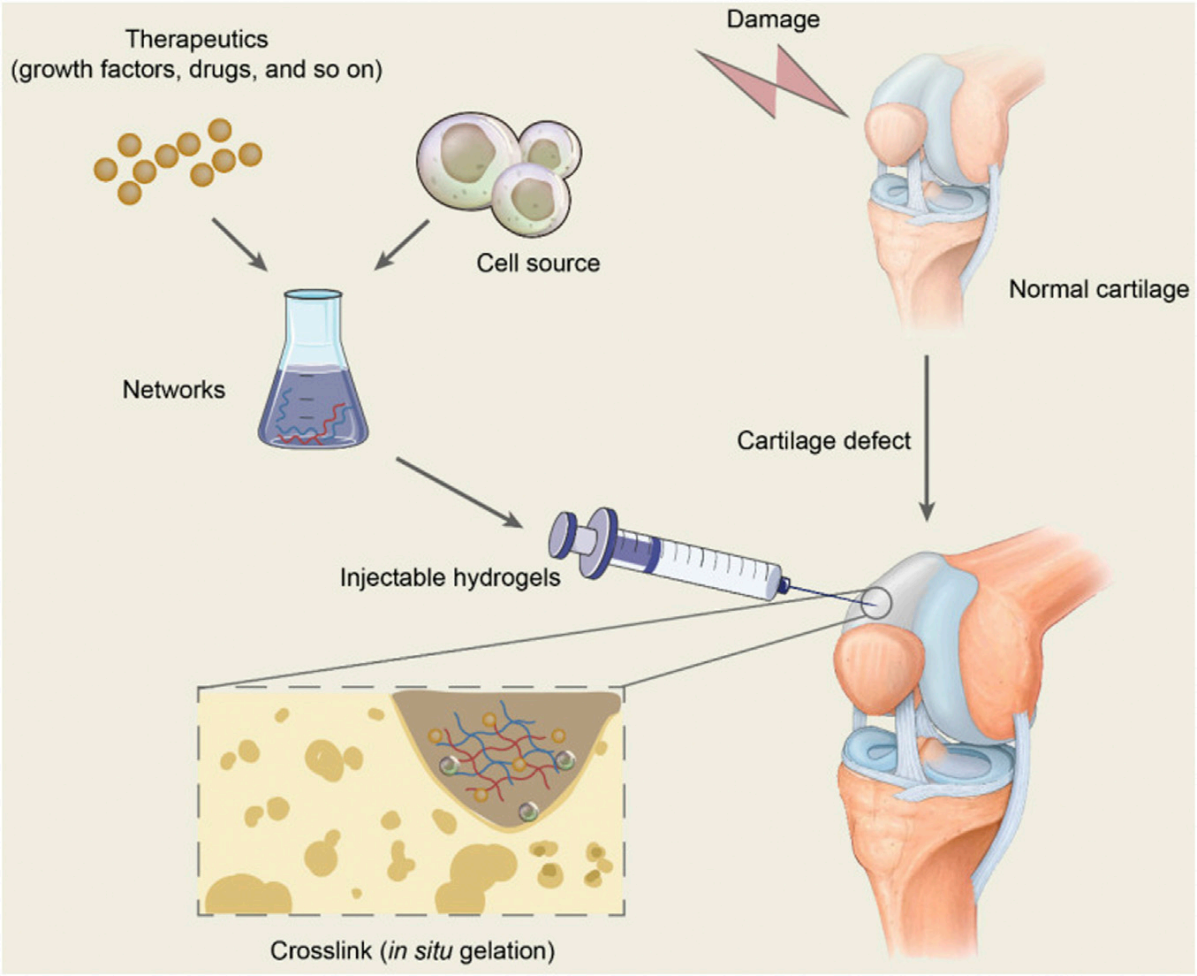
Schematic diagram of hydrogel injection in repairing cartilage defect. Adapted with permission of Wu et al. (2020).

Hydrogels are widely used in tissue engineering and are advanced cross-linked 3D hydrophilic polymer network biomaterials because of their unique properties such as high-water content, biocompatibility, porosity and biodegradation, solid elastic properties, deformability, and softness (Yang et al., 2017; Fu et al., 2018; Liu et al., 2021a). The hydrogel properties are similar to the characteristics of natural cartilage ECM and are easy to prepare. Hydrogel development is the most promising method for treating cartilage defects and cartilage regeneration (Wei et al., 2021; Huang et al., 2022). Injectable hydrogels have attracted the attention of biomaterial scientists in cartilage tissue engineering (Schaeffer et al., 2020; Wang et al., 2021a) (Figure 1). Because it can replace open implants with minimally invasive injections, it has the advantages of being less invasive, fewer complications, shorter hospital stays, and forming any desired shape *in situ* to match irregular defects (Liu et al., 2017a; Pascual-Garrido et al., 2018; Lin et al., 2021a). Injectable hydrogels provide hydration similar to the height of cartilage ECM. Biocompatibility and biodegradability of 3D structure and elastic properties can be controlled by improving cell metabolites and the supply of nutrients. The stimulus-response release mechanism can encapsulate cells and deliver efficient and effective bioactive molecules to the target of cartilage regeneration (Park et al., 2009; Pereira et al., 2009; Li et al., 2019). An ideal injectable hydrogel has several requirements, such as no toxic byproducts during *in vivo* gelation, appropriate solubility and gelation under physiological conditions, and a controlled gelation rate suitable for clinical practice (Jeznach et al., 2018).

This review aims to clarify the application of advanced injectable hydrogels in cartilage repair and regeneration. The progress and advantages of injectable hydrogels in cartilage repair and regeneration are reviewed, including the manufacturing technology (crosslinking method and structure) and suitable materials (polymers, cells and stimulators). Then, we summarize the research progress of the latest advanced injectable hydrogels in cartilage tissue engineering. Finally, the challenges in applying injectable hydrogels and their prospects in tissue engineering are also discussed.

### 1.1 Formation of injectable hydrogels

**FIGURE 2:**
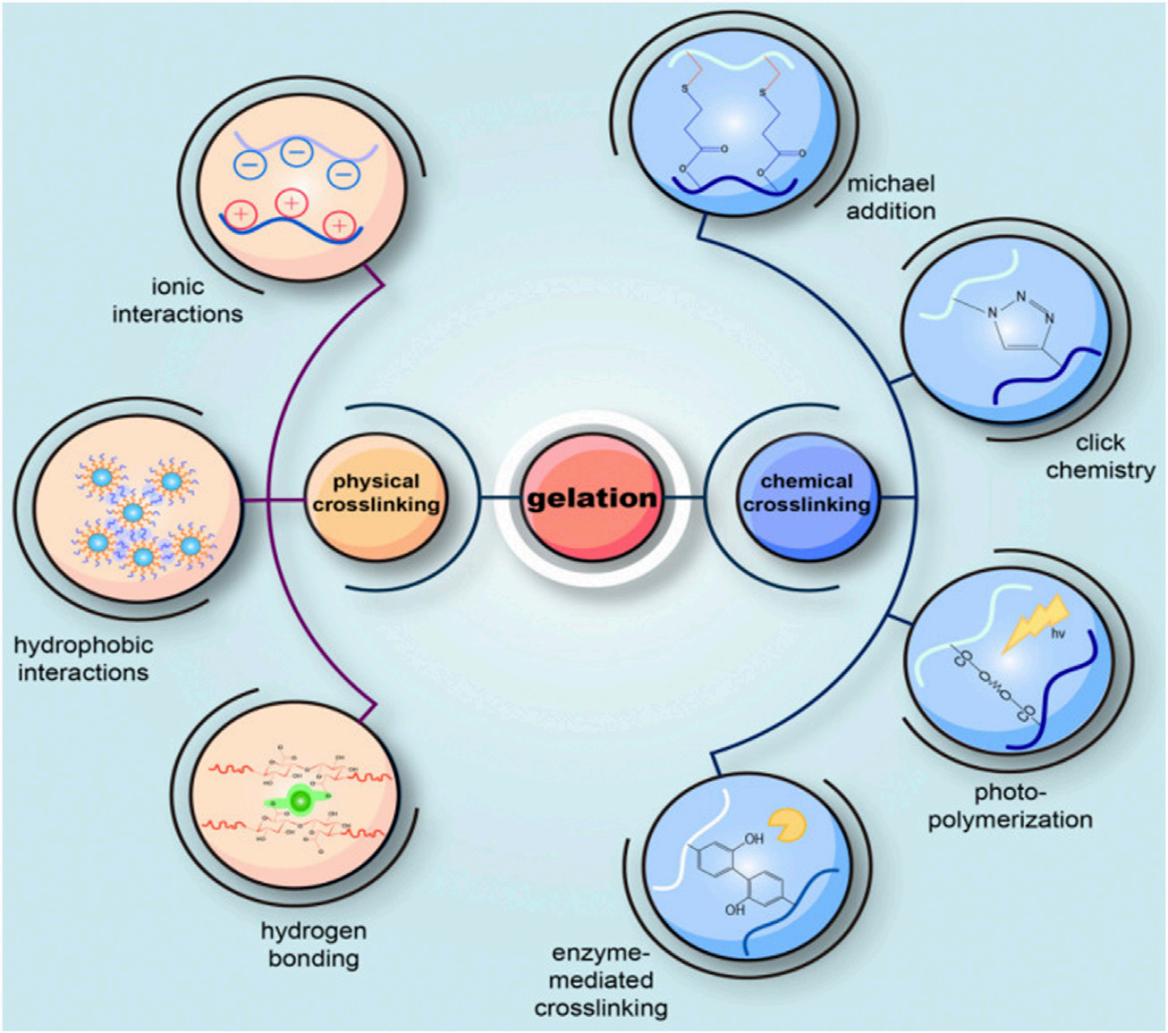
Schematic diagram of common methods for preparing injectable hydrogels. Injectable hydrogels can be roughly divided into two gel methods: physical cross-linking and chemical cross-linking reactions. The difference between them is whether or not covalent bonds are formed. Physical cross-linking is non-covalent bonding via reversible and instantaneous connections, including physicochemical or molecular entanglement interactions (hydrogen bonding, ionic or hydrophobic interactions). Chemical cross-linking forms covalent bonds in various chemical processes, including enzyme-mediated cross-linking, photopolymerization, click chemistry, Michael’s addition, Schiff base chemistry, and cross-linking agents. Adapted with permission of Wu et al. 2(2020).

Gelation is a crucial step in the formation of an injectable hydrogel. According to the design structure and standard application, it is imperative to select the appropriate formation method to prepare injectable hydrogels (Li et al., 2012). There are several ways of preparing injectable hydrogels based on their reactivity or the connections they contain. The cross-linking mechanism of the hydrogel can be divided into chemical cross-linking and physical cross-linking (Liu et al., 2020) (Figure 2). One of the distinctions between them is whether or not covalent bonds are formed (Wu et al., 2020).

### 1.2 Physical cross-linking

Hydrogels can be cross-linked *via* reversible networks or physical cross-links through physicochemical or molecular entanglement interactions, such as hydrophobic interactions, hydrogen bonds, ionic interaction, supramolecular chemistry, crystal formation, or charge condensation (Lu et al., 2018; Niemczyk et al., 2018). The mutual effects that occur in this hydrogel are fragile. However, they are numerous and lead to complex behaviors (Lynch et al., 2017; Bustamante-Torres et al., 2021; Muir and Burdick, 2021). Some injectable hydrogels by physical cross-linking are described below.

#### 1.2.1 Hydrophobic interactions

Hydrophobic interactions (also known as hydrophobic bonding) play a significant role in the self-healing course of soft materials (Tu et al., 2019). This interaction is stronger than the van der Waals and hydrogen bond interaction. Hydrophilic and hydrophobic parts are usual in molecules that form gels through hydrophobic interactions. Hydrophobic interactions are constituted between non-polar parts to reduce their contact with water (Skopinska-Wisniewska et al., 2021).

#### 1.2.2 Hydrogen bonding cross-linking

Hydrogen bonds can form cross-linking networks between hydrogen and electronegative atoms (He et al., 2019). Supramolecular hydrogels enhanced by multiple hydrogen bonds have good self-recovery, toughness, and recoverability as a driving force (Yu et al., 2021a).

#### 1.2.3 Ionic interaction

The cross-linked hydrogel structure is formed when molecules with opposite electric charges interact electrostatically (Abdulghani and Morouço, 2019). Ion interactions have been widely used to physically cross-link natural polysaccharides, such as chitosan and alginate, to prepare hydrogels (Huang et al., 2017). Alginate can gelate in the presence of polyvalent cations such as Sr^2+^, Ca^2+^, Fe^2+^, Co^2+^, and Ba^2+^, which is related to cation binding through G blocks of alginate and the formation of so-called “egg boxes” (Lee and Mooney, 2012; Abasalizadeh et al., 2020). CaCl_2_ is the most commonly used ion cross-linking agent in alginate hydrogel. Due to the high solubility of CaCl_2_ in aqueous media, alginate gelation rates are too fast to control. In addition, reduced gel rate results in greater mechanical integrity and a more uniform structure. CaCO_3_ and CaSO_4_ can be used instead to slow down the gelling speed. In addition, a buffer containing phosphate (such as sodium hexametaphosphate) can be used because the phosphate group in the buffer competes with the carboxylic group of alginate in the reaction with calcium ions, thereby reducing gelation (Abasalizadeh et al., 2020; Piras and Smith, 2020; Hu et al., 2021a). Cai et al. (2021). successfully prepared an injectable hydrogel by *in situ* cross-linking sodium alginate with divalent cations released from strontium-doped bioglass. The hydrogel’s biocompatibility and mechanical properties promoted BMSC proliferation, cartilage-specific gene expression, and glycosaminoglycan secretion.

#### 1.2.4 Supramolecular chemistry

Supramolecular chemistry hydrogels have been widely used in tissue engineering, bioelectronics, and drug delivery. It has good biocompatibility and biodegradation and contains many cell adhesion sites (Kim et al., 2011). As a key sense in supramolecular chemistry, self-assembly is mainly based on non-covalent interactions (hydrophobic/hydrophilic interactions, hydrogen bonding, van der Waals interactions, π-π stacking, and host-guest complexation) between molecules (Antoniuk and Amiel, 2016; Wang et al., 2020a). The substrate of supramolecular hydrogels, a basic molecular process, is usually non-covalent, structural, three-dimensional, responsive, dynamic, adaptive, and organized. Such molecular processes can easily interact with, interfere with, and even simulate cellular events in various biological systems (Zhou et al., 2017). Supramolecular interactions can promote physical cross-linking to form hydrogels in two ways. The first method is commonly used to create supramolecular materials, molecular gels made of small molecules with high aspect ratios, such as peptides. Once assembled, supramolecular stacks of small particles constitute long, typically rigid fibers. The second approach is that the interactions act as connections between polymer chains, including motifs based on host-guest complexation, metal-ligand ligands, and biomolecular binding (Mantooth et al., 2019; Zhou et al., 2020). Cyclodextrin-mediated host-guest interaction is an effective material for hydrogel construction, mainly because of its bioavailability, ease of chemical modification, and high reversibility and specificity of inclusion complexes composed of many hydrophobic guest molecules (Antoniuk and Amiel, 2016).

### 1.3 Chemical cross-linking

The convergence of years of research has led to the development of mild chemistry that can be set at temperature, physiology, osmotic pressure, and pH while avoiding using toxic reagents. Chemical cross-linking depends on the formation of covalent bonds between the reactive groups grafted to the main chain of the polymerization, and it can occur under certain conditions (Flégeau et al., 2017). These conditions include click chemistry, Michael addition, disulfide cross-linking, enzyme-mediated cross-linking, silanization, Schiff base chemistry, photopolymerization, and cross-linking agents (Radhakrishnan et al., 2014; Piantanida et al., 2019; Nguyen et al., 2021)

#### 1.3.1 Click chemistry

Click chemistry includes copper-catalyzed alkyne-azide cycloaddition, copper-free click (strain-promoted azylene cycloaddition click, Diels-Alder click chemistry, oxime, mercaptan, and mercaptan alkyne), and pseudo click (Gopinathan and Noh, 2018; Li et al., 2021a). Click chemistry is widely used in constructing injectable hydrogels due to its mild reaction conditions, high chemical selectivity, and fast gelation time, without adding or producing cytotoxic cross-linking agents, chemical additives, and byproducts in the gelation process (Yao et al., 2020).

#### 1.3.2 Michael addition

Michael addition reaction hydrogels are prepared by adding polymers containing thiol groups to α, β-unsaturated carbonyl polymers under standard conditions (Quadrado et al., 2021). PEG-based hydrogels based on the Michael addition reaction have been widely used in tissue engineering (Guo et al., 2021). Pupkaite et al. (Pupkaite et al., 2019) tried to overcome the shortcomings of partially injectable hydrogels, such as complex, overexpanding, potentially toxic cross-linking processes, or lack of self-healing and shear thinning. Mercaptan groups were introduced into collagen. The hydrogel was prepared by cross-linking with 8-arm polyethylene glycol maleimide. The hydrogel is cytocompatible and can be used to encapsulate and deliver cells.

#### 1.3.3 Enzyme-mediated cross-linking

Enzyme cross-linking reactions are mild. Most enzymes catalyze reactions in water environments at neutral pH and moderate temperatures. This means that they can also be used to form hydrogels *in situ*, avoiding the loss of biological activity caused by natural polymers that cannot withstand the harsh chemical conditions required for crosslinking (Teixeira et al., 2012). Several enzyme-mediated injectable hydrogels are used for cartilage defect repairs, such as tyrosinase, lysyl oxidase, and transferase enzyme systems (Liu and Lin, 2019; Wang and Wang, 2021). HRP and H_2_O_2_ are the most common enzyme-mediated cross-linking agents by phenol partial carbon-oxygen/nitrogen bond or carbon-carbon bond oxidative coupling. They can easily control the physical properties of the hydrogel by changing their concentration (Ren et al., 2015). These hydrogels are formed in a matter of minutes. They showed excellent biocompatibility and supported chondrocyte proliferation and differentiation (Jin et al., 2010). Enzyme-mediated hydroxyapatite hydrogel has the advantages of injectable, non-cytotoxic, and rapid cross-linking (Jin et al., 2020). Zhang and his team proposed a biomimetic enzyme complex of ferrous glycine (Fe [Gly]_2_) and glucose oxidase for rapid (less than 5 s) and mild preparation of injectable tough hydrogels (Zhang et al., 2021a).

#### 1.3.4 Schiff base chemistry

Schiff base chemistry involves the formation of dynamic covalent imine bonds by cross-linking aldehyde and amine groups. Schiff base chemistry has the advantages of being reversible, simple, pH-sensitive, and biocompatible (Xu et al., 2019; Sahajpal et al., 2022). For the formation of biopolymer hydrogels, the functions of hydrazones and imines are most commonly used to achieve dynamic cross-linking behavior (Muir and Burdick, 2021). Chen et al. (2021). prepared injectable HA hydrogels modified with methacrylate and aldehyde group through dynamic Schiff base reaction. The results showed that the hydrogel was easy to prepare quickly *in situ*, had good biocompatibility, promoted BMSC proliferation, and promoted the regeneration of rat cartilage.

#### 1.3.5 Photopolymerization

Visible or near ultraviolet photopolymerization is one of the most widely studied gelation processes in the development of injectable hydrogels. Some types of hydrogels can be photopolymerized *in vitro* and *in vivo* by the interaction of photoinitiators with visible or ultraviolet light to generate free radicals and polymerize free radical chains (Meng et al., 2019; Wu et al., 2020). Photopolymerization is a fascinating method with the following characteristics: 1) It is based on chemical reactions unaffected by water, making it suitable for use in aqueous media. 2) This is usually a very rapid process, allowing the synthesis of free-standing hydrogels in minutes or seconds. 3) It allows space and time control of the cross-linking process. 4) It is very little cytotoxic under the appropriate conditions and thus does little harm to cell survival and proliferation (Nicol, 2021; Pierau and Versace, 2021). The researchers altered collagen with Methacrylamide to photo-crosslinking under ultraviolet stimulation, enabling fast *in situ* gelation (Zhang et al., 2020a). GelMA injectable hydrogel is formed by introducing a double bond into a gelatin polymer chain that rapidly forms a hydrogel under light initiation. The blue light initiator lithium phosphonate makes the gelation approach faster and the preparation approach easier (Yue et al., 2015; Wang et al., 2021b; Wang et al., 2021c).

### 1.4 Comparison of physical and chemical cross-linking

The ideal injectable hydrogel has several requirements, including: 1) no evil byproducts are produced after gelation; 2) solubility of the gelated aqueous solution under physiological conditions (pH, temperature, and ion concentration); 3) the rate of gelation is rapid enough to meet the clinical efficacy. Nevertheless, in the presence of an additional agent such as a cell or bioactive molecule, there is adequate time for appropriate mixing and injection; 4) Suitable rate of biodegradation (Salavati-Niasari and Davar, 2006; Naahidi et al., 2017; Elkhoury et al., 2021). Both physical and chemical gelation must fulfill the above requirements. However, both physical and chemical techniques have benefits and deficiencies. Therefore, the most suitable method should be selected to design injectable hydrogels.

Compared to chemical cross-linked hydrogels, physically cross-linked hydrogels typically exhibit lower mechanical properties because the physical interactions are reversible and weak, so the hydrogels that form loosen easily when physical conditions (temperature, ionic strength, electrolyte, and pH) change (Mathew et al., 2018). For example, thermosensitive cross-linked hydrogels are one of the most widely studied injectable hydrogels in tissue engineering (Xu et al., 2020; Torres-Figueroa et al., 2021). Sol-gel transformations occur during hydration and dehydration at different temperatures (Shi et al., 2021a). The CST of such hydrogels is close to the body temperature during the sol-gel transition. The polymer chain expands in a random coil conformation due to its hydrophilic interaction with water molecules. However, when the system is heated beyond CST, the polymer chains collect and collapse due to a major hydrophobic interaction between the polymer chains (Sala et al., 2017; Dethe et al., 2022). Different PEG-based polymers, Poloxam and NIPAAm, are typical examples of thermosensitive hydrogels. The polar groups in the hydrogel can form hydrogen bonds with the water molecules between the polymer chains under CST, making it soluble. Above CST, the polymer chain contracts and becomes insoluble and hydrophobic, resulting in gelation (Eslahi et al., 2016).

Furthermore, ion-sensitive injectable hydrogels for cartilage defect repair have been developed. By adding Ca^2+^, the alginate solution can easily constitute hydrogel through ion cross-linking through the calcium bridge between the guluronic acid residues on nearby chains (Hu et al., 2019). pH-responsive hydrogels consist of polymers with basic or acidic groups, and their mechanisms involve dissociation and binding with hydrogen ions in response to ambient pH. This hydrogel has been extensively studied in drug delivery applications because the pH curves of pathological tissues (such as infection, inflammation, and cancer) differ from those of normal tissues (Eslahi et al., 2016; Yu et al., 2020a).

These physically cross-linked injectable hydrogels can be converted from liquid to gel and organized *in situ* hydrogels when injected into the body without additional cross-linking agents, chemical reactions, or environmental treatments (Gao et al., 2020). Physical interactions (e.g., hydrogen bonds, electrostatic attraction) or reversible chemical bonds (e.g., imine bonds) can form cross-linked pre-gel hydrogels whose structures are reversible (Arkenberg et al., 2020; Gupta et al., 2020). Pre-gelated hydrogels are in vitro-formed hydrogels that can be injected at the target and self-heal (Oliva et al., 2017). Pre-gelated hydrogels are injectable due to their self-healing and shear thinning (Riley et al., 2019). As the shear rate increased (during injection), the stickiness of the hydrogel decreased dramatically, reflecting the shear-thinning behavior (Thambi et al., 2017; Wang et al., 2021d). Although the injection forces may interfere with the cross-linking structure and trigger the gel-sol transition, the following self-healing process can rebuild the gel after removing the strain (Liu and Hsu, 2018). Shear-thinning injectable hydrogels protect encapsulated cells from high shear forces, improving the effectiveness of cell-based therapies (Thakur et al., 2016).

On the other hand, chemically cross-linked gels typically have stronger mechanical properties because covalent bonds are permanent and rigid (Zhao et al., 2020; Ali et al., 2022). The main drawback of chemical cross-linked hydrogels is the problem of cytotoxicity, which binding reactive compounds and light radiation may cause. Fortunately, recent developments in chemical cross-linking methods have enabled good biocompatible hydrogels to be gelated under mild reaction conditions (Lee et al., 2020; Wu et al., 2020).

Advanced injectable hydrogel preparation methods need to be further developed to improve physiological stability and mechanical properties, reduce adverse effects and cytotoxicity of hydrogels *in vivo*, and ensure gelation occurs at a rate suitable for clinical practice. Each approach has its advantages and disadvantages. Future research will explore how to correctly select the appropriate method and improve the existing manufacturing method.

### 1.5 Multiscale structure of injectable hydrogels

**FIGURE 3:**
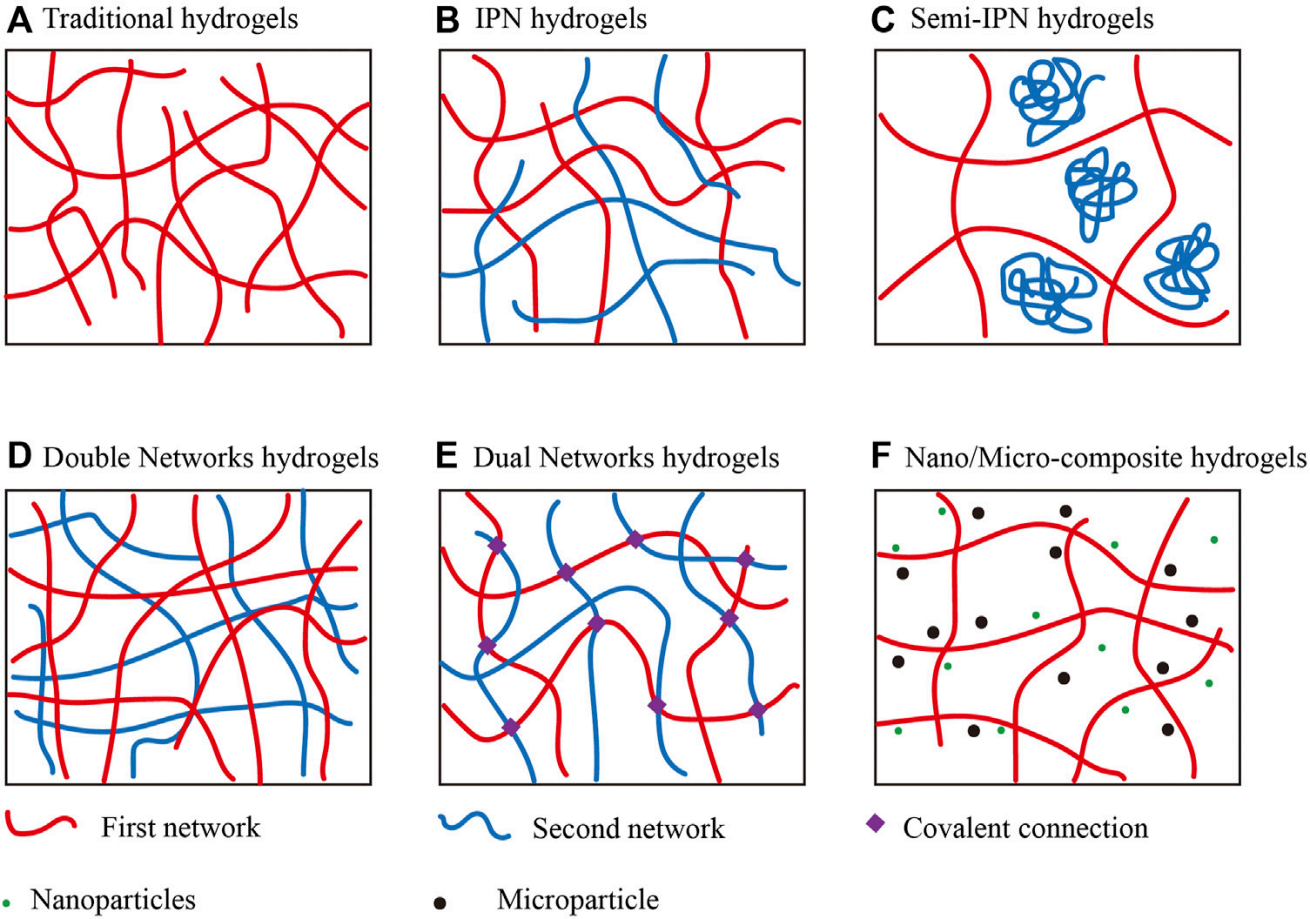
The schematic diagram depicts injectable hydrogels with different structures for cartilage regeneration engineering **(A)**. Traditional single polymer networks. **(B–F)**. Different multiscale structure of injectable hydrogels. Reproduced with permission of Vega et al. (2017).

The bearing capacity of materials is a crucial characteristic in cartilage tissue engineering. Cartilage reduces friction, shear, and compression forces between bones. Its modulus is 0.5–2 MPa (Cross et al., 2016). Hydrogel has a stiffness of two orders of magnitude lower than natural cartilage (Li et al., 2019). Poor mechanical properties and limited functionality of traditional injectable hydrogels hinder their application in cartilage (Song et al., 2015). In addition to high water content and biocompatibility, the rigid multiscale hydrogel system also has super tensile property and large fracture energy (Xin, 2022). Multiscale injectable hydrogels with high mechanical strength and stability are of particular interest in cartilage tissue engineering (Figure 3).

### 1.6 Interpenetrating polymer network hydrogels

The IUPAC defines IPN as a unique polymer mixture consisting of two or more cross-linked networks whose parts are intertwined but not covalently connected and which cannot be separated unless the chemical bond breaks (Matricardi et al., 2013). IPN hydrogel was exploited to improve its mechanical properties (Zhang et al., 2015). Compared with hydrogels formed by a single polymer model, hydrogels with IPN often exhibit superior mechanical properties (Zoratto and Matricardi, 2018). Shojarazavi et al. (2021) used an IPN structure combined with silk fibroin nanofibers, alginate, and sodium cartilage ECM to enhance the mechanical properties of ECM to achieve the mechanical stiffness required for cartilage repair.

### 1.7 Semi-interpenetrating polymer network hydrogels

Unlike IPN, the chains of the second type of polymer in Semi-IPN are only dispersed in the network formed by the first type of polymer, rather than forming another network interpenetrating with the first type of polymer (Aminabhavi et al., 2015). IUPAC defined Semi-IPN as a polymer consisting of one or more networks and branched or linear polymers characterized by the penetration of at least one network at the molecular scale by at least some branched or linear macromolecules (Rinoldi et al., 2021). Furthermore, branched or linear polymers composed of Semi-IPN can be separated from the composed polymer network without breaking the chemical bond (Dhand et al., 2021). Thermosensitive hydrogels based on the chitosan/β-glycerophosphate system are widely used in cartilage regeneration engineering because of their good injectable properties, rapid gelation at the injection site, and ability to repair cartilage defects (Saravanan et al., 2019). However, due to their physical cross-linking network, chitosan/-glycerophosphate hydrogels exhibit high degradation rates and poor mechanical properties under physiological conditions, limiting their application (Jalani et al., 2015; Saravanan et al., 2019). Panyamao et al. (2020) used GE covalent cross-linking and pullulan Semi-IPN to improve the mechanical properties and swelling capacity of injectable thermosensitive hydrogels ground on the chitosan/β-glycerophosphate organization. Moreover, Wang et al. (2020a) prepared an injectable Semi-IPN hydrogel based on HA-SH and BPAA-AFF-OH supramolecular short peptides. The injectable hydrogel exhibits reliable mechanical strength. Moreover, compared with HA-SH hydrogel, it can enhance the expression of chondrogenesis-related genes and matrix secretion and further promote the maintenance of the hyaline cartilage phenotype.

### 1.8 Double networks hydrogels

Double-network hydrogels consist of two cross-linked networks with significantly different mechanical properties. The first network provides a rigid structure, and the second network is malleable. This is due to some structural parameters, namely the rate of the two hydrogel components, cross-linking density, swelling rate, and molecular weight allocation of the network polymer (Jonidi Shariatzadeh et al., 2021; Xin, 2022). Wang et al. 2(2021b) studied a double injectable hydrogel based on HAMA and GelMA. The double hydrogel combines the strong mechanical properties of HAMA with GelMA’s role in chondrocyte phenotype maintenance and ECM formation.

### 1.9 Dual networks hydrogels

Unlike a double network using two different mechanical properties materials, a dual network is defined as two cross-linked materials to form the same network and have a similar cross-linking mechanism. Although the dual network does not have the toughness of the double network, each material in the dual network can inject other useful properties into the hydrogel. For example, one material attracts cells and encourages migration into the injectable hydrogel, while the other effectively binds to surrounding tissue (Vega et al., 2017). Kim et al. (2021). (investigated poly(N‐isopropyl acrylamide) and PLL-based dual network injectable hydrogels encapsulating articular chondrocytes and MSCs. The model experiment of cartilage transplantation *in vitro* showed that dual hydrogel could promote cartilage defect repair.

### 1.10 Nano/micro-composite hydrogels

Mixed hydrogels integrated with nano/micron composites are networks of hydrated polymers physically or chemically cross-linked with N/MPs or other nano micron structures (Mehrali et al., 2017; Kouser et al., 2018).

N/MPs have excellent mechanical properties, surface reactivity, bioavailability, and a larger surface-to-volume ratio (Asadi et al., 2018; Zinatloo-Ajabshir et al., 2018; Piantanida et al., 2019; Ahmadian-Fard-Fini et al., 2020). The hard N/MPs enhance the soft organic polymer matrix, and the resulting nano/micron composites hydrogel can exhibit novel or enhanced biological, mechanical, conductive, optical, or magnetic properties (Motahari et al., 2015; Motealleh and Kehr, 2017; Zhao et al., 2017; Ravari et al., 2021). Inorganic materials such as clay, graphene, CNCs, hydroxyapatite, and metal nanoparticles can be used as fillers to enhance the hydrogel matrix (Davar et al., 2010; Zazakowny et al., 2016; Asadi et al., 2018; Zinatloo-Ajabshir and Salavati-Niasari, 2019). Nano-silicates with high cellular and biocompatibility could form shear-thinning hydrogels when combined with long-chain polymers (Thakur et al., 2016; Lokhande et al., 2018). POEGMA precursor polymer was physically cross-linked with CNCs, which made CNCs have excellent hydrogel dispersibility and significantly enhanced gel mechanical properties. Other gel properties, including swelling, degradation kinetics, and gelation rate, also changed significantly (De France et al., 2016). GelMA injectable hydrogel microspheres prepared by microfluidic technology are widely used in cartilage repair due to their enhanced high injectivity, structural stability, and uniform size (Han et al., 2021). Lei and his team reduced articular cartilage friction by coating the surface of injectable hydrogel microspheres with liposomes to form a self-renewing hydration layer through friction and wear. In addition, the release of an autophagy activator (rapamycin) promotes cartilage repair (Lei et al., 2022).

Moreover, the N/MPs adsorbs and retain essential stimulating factors, prolonging their release time due to the larger surface-to-volume ratio and high encapsulation efficiency of stimulating factor (Amiri et al., 2017; Nagahama et al., 2018; Wong et al., 2018; Ishihara et al., 2019; Bian et al., 2021; Zewail et al., 2021; Luu et al., 2022; Seo et al., 2022). Wang et al. (2020b) loaded the water-soluble antibiotic isoniazid into a cross-linked PEG network and encapsulated the hydrophobic antibiotic rifampicin into mesoporous silica nanoparticles. The addition of nanoparticles can significantly adjust and enhance injectable hydrogels' mechanical strength and elasticity. The release time of rifampicin was significantly longer than isoniazid and promoted cartilage repair. Lin et al. (2021b) developed PLGA microspheres loaded with TGF-β3, and injectable hydrogel-coated PLGA microspheres could sustainably release TGF-β3. This synthetic micron composite injectable hydrogel system regulates chondrocyte differentiation and biosynthesis.

The electroactive nanomaterials promote the migration, adhesion, proliferation, and differentiation of preosteoblasts and MSCs. Aniline oligomers (Penta aniline or tetra aniline) are the most commonly used conductive oligomers, with the advantages of good biocompatibility, low cost, easy synthesis, good stability, easy processing, electrochemical behavior similar to conductive polymers, and due to low MW, easy to be eliminated from the body by renal excretion (Wang et al., 2016; Hassanpour et al., 2017; Zinatloo-Ajabshir et al., 2020; Monsef and Salavati-Niasari, 2021).

## 2 Material of injectable hydrogels

Injectable hydrogels are generally required to have the following characteristics, including low toxicity, adequate biocompatibility, support for cell adhesion, proliferation and differentiation, biodegradability, appropriate degradation rate, and fine structure similar to the tissue or organ to be repaired, and controlled release of biomolecules (Jiang et al., 2021). Strategies for cartilage repair based on natural and synthetic injectable hydrogels and their combination were studied.

### 2.1 Natural polymers

Natural polymers can be broadly divided into three categories: those based on proteins, polysaccharides, and protein nucleotides. Because ECM is a complex combination of fibrin (such as laminin, collagen, and ficonin) and hydrophilic proteoglycan. A simple and effective way to simulate ECM is to prepare injectable hydrogels using natural polymers that mimic many of its characteristics (Farrell et al., 2017). Natural polymers have several advantages over synthetic polymers:1) they are more biocompatible; 2) They can contain cell-binding motifs, realize cell adhesion, proliferation, and biological activity cues, and influence cell behavior; 3) They can exhibit fibrous structures that mimic the ECM of natural tissues; 4) They can be recognized and processed metabolically by the body, allowing cells to reshape them along with cell-secreted ECM deposition (Gomez-Florit et al., 2020). On the other hand, natural polymers have low mechanical strength and vary from batch to batch and from natural source to natural source, making their molecular weight, chemical structure, and rate of degradation difficult to control (Yan et al., 2014; Coenen et al., 2018).

### 2.2 Protein polymer

#### 2.2.1 Silk fibroin

SF polymers can be easily processed into various forms, including micron/nanoparticles, membranes, films, fibers, and mainly hydrogel scaffolds (Zheng et al., 2022). Chemical and physical methods can prepare SF injectable hydrogels through the β-sheet formation. Various chemical reagents, including alcohols, acids, salts, and surfactants, have been used as SF crosslinkers (Yuan et al., 2021a).

#### 2.2.2 Gelatin

Gelatin is a commercial biomaterial whose biological properties are widely used in biomedical engineering due to its similarity to more expensive collagen as an adhesive protein (Tonda-Turo et al., 2017). Gelatin is produced by regional hydrolysis of collagen and can promote cell adhesion, proliferation, migration, and differentiation due to RGD sequence in its structure (García-Fernández et al., 2020). In addition, one of the main characteristics of this water-soluble protein is its thermal response, with reversible sol-gel transformations occurring when cooled above the critical solution temperature (25–35°C). Gelatin is therefore widely used in cartilage tissue engineering (Echave et al., 2019).

#### 2.2.3 Collagen

Two-thirds of the dry weight of the adult joints cartilage is collagen. The fibrous network of developing cartilage is a copolymer of collagen XI, IX, and II and small amounts of other types of collagen (Eyre, 2004). Varieties of combinations of collagen with other natural polymers like inulin, combinations of fibrin, alginate, and gelatin have been transformed into formulations for injectable hydrogels, enabling *in-situ* formation (Rigogliuso et al., 2020).

### 2.3 Polysaccharide polymer

#### 2.3.1 Alginate

Alginate is a polysaccharide extracted from brown seaweed and composed of α-l-guluronate (G block) and β-D-mannuronate (M block) copolymers linked by 1, 4-glycosidic bonds. G block of alginate is cross-linked with divalent cations such as Ba^2+^ and Ca^2+^ to form gel (Balakrishnan et al., 2014). It is widely used due to its biocompatibility, biodegradability, and ease of manufacture. However, alginate lacks the property of cell adhesion. Alginate was mixed with other polymers for cartilage repair to improve its biological properties (Jaikumar et al., 2015).

#### 2.3.2 Chitosan

Chitosan natural polymer is a widely available polysaccharide created by completely deacetylating chitin, a structural component extracted from insect and crustacean bones. Importantly, chitosan-based materials have received much attention as hydrogels because of their good cellular compatibility, pH sensitivity, and biodegradability. In general, chitosan could dissolve in acidic solutions, and its viscosity properties can be easily adjusted by adjusting the concentration (Zheng et al., 2022). The insolubility of chitosan in water limits its use. Therefore, many studies have focused on soluble chitosan derivatives (Fattahpour et al., 2020). For example, chitosan becomes a thermosensitive polymer when mixed with polyol phosphate salts like β -glycerophosphate (BGP) (Panyamao et al., 2020).

#### 2.3.3 Hyaluronic acid

HA is the primary component of glycosaminoglycan in ECM. It consists of repeated disaccharide units of n-acetyl-D-glucosamine and β -D-glucuronic acid, alternately linked by β-1,4 and β-1,3 glycosidic bonds (Graça et al., 2020; Li et al., 2021b). Natural HA does not affect cell adhesion or gel formation. Hence, it is necessary to chemically alter the functional groups of HA, adjusting their physical, chemical, or biological properties according to special requirements of specific applications (Zaviskova et al., 2018). In the absence of chemical cross-linking agents, hydrogels are formed by Schiff bases between the amino group of ethylene glycol-chitosan and the aldehyde group of oxidized hyaluronate. These hydrogels show good bio durability and compatibility under physiological cases, and they may be a potential injectable cell delivery system in cartilage tissue engineering (Kim et al., 2017).

#### 2.3.4 Agar

Agar is a water-extracted cell-wall polysaccharide from Gracilariaceae and Gelidiaceae plants of seaweeds, consisting mainly of (1–3) 3, 6-hydroxy-lactose repeated units and alternating (1–4) D-galactose. Agar is solvable in water at temperatures beyond 65°C and forms a gel between 17 and 40°C (Tonda-Turo et al., 2017). Agar and gellan gum promote cartilage regeneration by inhibiting inflammatory mediators and inducing chondrogenesis and autophagy-related gene expression (Heo et al., 2020).

#### 2.3.5 Gellan gum

Gellan gum is a bacterial polysaccharide extracted from Sphingomonas elodea. Its main glycoside chain is a repeating tetrasaccharide unit, each repeating unit contains one acetate and one L-glyceride, and one esterified substituent occurs in every two sequences (Oliveira et al., 2021). Gellan gum can form a thermally reversible injectable gel with no cytotoxicity in different test environments. It is commonly used in the food industry and has previously been used for drug delivery in the biomedical field. Gellan gum can effectively regenerate hyaline cartilage tissue in the defect (Oliveira et al., 2010). Choi et al. (2020) loaded 6-(6-amino-hexyl) amino-6-deoxy-β-cyclodextrin onto the Gellan gum chain to reduce gel temperature, enhance physicochemical properties, and improve drug delivery efficiency and release.

#### 2.3.6 Cellulose

The physical capabilities of cellulose depend on the presence of three hydroxyl groups (OH) at the C-6, C-3, and C-2 positions. Cellulose injectable hydrogels made from carboxymethyl cellulose (CMC), methylcellulose (MC), and hydroxypropyl cellulose (HPC) have good mechanical properties and are biocompatible (Zhang et al., 2021b).

#### 2.3.7 CS

CS is a GAG consisting of alternating units of (β-L, 4) n-acetylgalactosamine (GalNAc) and β-1,3-linked glucuronic acid (Glca). The residues of galactosamine at position 4 or 6 can be sulphated (Yuan et al., 2021b). Furthermore, CS is the most abundant GAG in the human body and the main component of chondrocyte ECM, which has attracted great attention as a biomaterial for cartilage defect repair. CS in cartilage has multifold key roles, including supporting chondrogenesis, providing resistance to stress, chondrocyte signaling, and intercellular communication (Thomas et al., 2021).

### 2.4 Protein nucleotide polymer

#### 2.4.1 Deoxyribonucleic acid

DNA is a brilliant molecule because of its biocompatibility, minimal toxicity, precise molecular recognition, and easy programming (Li et al., 2020a; Li et al., 2022). Physical tangles between DNA strands or chemical connections between DNA molecules can be used to create DNA hydrogels. Chemically, polymers are held together by covalent bonds, which confer great mechanical strength and environmental stability (Kahn et al., 2017; Zhang et al., 2020b; Khajouei et al., 2020). DNA injectable hydrogels are widely used in cartilage repair engineering due to their injectable properties, adjustable mechanical properties, and good permeability (Yan et al., 2021).

### 2.5 Extracellular matrix

ECM hydrogel provides cells with a natural adhesion surface and superior biological activity. Preparing acellular ECM hydrogels can maximize the retention of growth factors and low molecular weight peptides present in natural ECM (Gong et al., 2021). At present, bionic and tissue-specific injectable hydrogels are prepared from various acellular ECM (amniotic membrane, cartilage, bone, heart, and lung) for cartilage regeneration engineering (Bhattacharjee et al., 2020; Gong et al., 2021; Bhattacharjee et al., 2022). Bordbar et al. (2020) developed an injectable hydrogel derived from acellular sheep chondrocyte ECM. The cells embedded in the hydrogel can differentiate into chondrocytes. Sevastianov et al. (2021) compared the effects of injectable collagen hydrogels and acellular porcine articular cartilage injectable hydrogels on rabbit BMSCs differentiation. Injectable collagen hydrogel is more beneficial in stimulating BMSCs to repair cartilage *in vivo*, and injectable porcine articular cartilage is an inducer for BMSCs to form chondroid tissue *in vitro*.

### 2.6 Synthetic polymer

Synthetic polymer hydrogels have been developed to meet the need for more alternative materials in tissue engineering. Synthetic polymers mainly include polymers based on PLA, PGA, PLGA, PCL, PVA, and polyester copolymers (Werkmeister et al., 2010; Yan et al., 2014). Synthetic polymers of glycopeptides mimic natural glycoproteins or glycopeptides and have great potential in biomedical applications. The extracts of glycopeptide copolymer and glycopeptide hydrogel showed good cytocompatibility *in vitro*. When injected subcutaneously into rats, glycopeptide hydrogels formed rapidly *in situ* (Ren et al., 2015). A one-component synthetic methacrylate type II collagen can be photo-crosslinked to form a firm injectable hydrogel. MSCs encapsulated in this hydrogel showed good activity and could coagulate and undergo chondrogenesis (Behan et al., 2022).

#### 2.6.1 Poly lactic-co-glycolic acid

PLGA is a synthetic polypeptide formed by natural l-glutamic acid through an amide bond, biodegradable, avoids antigenicity or immunogenicity, and is non-toxic and hydrophilic. In addition, abundant side-chain carboxyl groups on the PLGA chain enable it to undergo chemical modification. These properties make PLGA an ideal biomedical material (Yan et al., 2014).

#### 2.6.2 Polyethylene glycol

PEG is a non-toxic, non-immunogenic, and pollution-resistant synthetic polymer widely used as a substrate in tissue engineering, such as articular cartilage, bladder, and nerve tissue regeneration (Li et al., 2021c). A PEGDA hydrogel involved in chondroitin sulfate binder has entered clinical trials for repairing cartilage defects and has shown improved results in combination with microfractures (Qi et al., 2018; Qi et al., 20182021).

#### 2.6.3 Polyglycolic acid

PGA is a polypeptide secreted by Bacillus subtilis natto. Many carboxylic acid groups (-COOH on the side chain of -PGA) are easily functionalized to achieve precise functions. The ultimate degradation product of PGA is glutamate, a component of collagen. Due to its excellent biocompatibility, biodegradability, and non-toxicity, PGA is used to prepare hydrogels with various functions for tissue engineering matrix, especially cartilage (Wei et al., 2022).

### 2.7 Natural/synthetic polymer

Synthetic polymers are easy to manufacture and replicate. However, they have poor biodegradability and biocompatibility. Naturally derived polymers are widely used in injectable hydrogels for repairing cartilage defects due to their excellent biodegradability, biocompatibility, and similar 3D microenvironment *in vivo*. However, rapid degradation, poor mechanical properties, and enhanced microenvironment for cell proliferation and differentiation are challenges in practical applications (Jian et al., 2012; Lee et al., 2021). The combination of natural and synthetic polymers can play to their respective strengths and compensate for their weaknesses (Peng et al., 2019; Li et al., 2021c). Yang et al. (2021) mixed injectable hydrogels of 2%DF-PEG/1.5%GCS, which significantly improved the mechanical properties and biocompatibility of hydrogels, and the hydrogels loaded with ADSCs promoted cartilage repair. Shi’s team researched and prepared an injectable hydrogel with natural antioxidant capacity. A dynamic covalent bond between PVA and phenylboronic acid grafted to HA-PBA forms hydrogels, which are further stabilized by secondary cross-linking between the acrylate portion of the HA-PBA and the free sulfhydryl group in the vulcanized gelatin (Shi et al., 2021b). The existence of a dynamic covalent bond contributes to the shear thinning of hydrogels, which makes hydrogels have suitable printing. Hydrogels protected coated chondrocytes from ROS-induced upregulation of Chondrocyte-specific catabolic genes (MMP13) and downregulation of anabolic genes (COL2 and ACAN) after incubation with H_2_O_2_.

### 2.8 Cells and stimulating factors integrated into injectable hydrogels

Injectable hydrogels can integrate appropriate cells and stimulating factors to stimulate damaged tissue’s original microenvironment and thus help regenerate damaged cartilage (Yang et al., 2017; Ngadimin et al., 2021). Injectable hydrogels act as the matrix to promote cell-cell interactions and cell-matrix interactions, while stimulating factors are part of signals that mediate cell adhesion and migration to scaffolds. Hence, cells and stimulating factors are important for applying injectable hydrogels in tissue engineering, as they play a vital role in cell differentiation and tissue growth (Sun et al., 2017; Cho et al., 2020; Stampoultzis et al., 2021). Living tissue cells migrate from the surrounding to the hydrogel and interact within the hydrogel to reconstruct the desired tissue at the implant site. Injectable hydrogels can also transport cells that interact with protocell populations and deliver growth factors or other therapeutic biomolecules to recapture abnormal biology (Dimatteo et al., 2018).

### 2.9 Cell source and cell capsulation

The requirements of injectable hydrogel-encapsulated cells for cartilage repair are as follows: 1) they can constitute cartilage tissue; 2) suitable for clinical application, that is, the source is vast, the trauma is minor, and the extraction is easy; 3) after many passages, they can obtain the required number of cells while maintaining the cartilage phenotype (Kwon et al., 2019). Embedding cells into injectable hydrogels can be achieved by embedding cells during gel formation or by inoculating cells into prefabricated porous gels (Armiento et al., 2018; Jabbari and Sepahvandi, 2022).

#### 2.9.1 Chondrocytes

ACI has been successfully used to promote articular cartilage regeneration. Hu et al. 2(2021b). loaded chondrocytes in IPN injectable hydrogel composed of chitosan/HA/Si-HPMC. The chondrocytes proliferated well *in vitro*, promoting cartilage defect repair in rat models *in vivo*. Chiang et al. (2021) prepared an injectable HA-PAA hydrogel with magnetic navigation and glutathione release. The chondrocytes embedded in the hydrogel proliferated and differentiated at the site of cartilage damage through magnetic interaction of internal iron nanoparticles and adhesion of CD44 receptors on HA chain. The rabbit cartilage defect model produced uniform and smooth, regenerated cartilage 8 weeks after hydrogel implantation, and the columnar arrangement of chondrocytes in the deep tissue was similar to that of normal chondrocytes (Chiang et al., 2021).

However, some shortcomings still need to be addressed, such as the low number of cells that are difficult to extract and harvest. With the increase of amplification generations, chondrocytes lose their chondrogenic phenotype. Eventually, this results in lower cartilage repair (Cai et al., 2020; Zhou et al., 2022). Autologous chondrocytes are mainly derived from natural cartilage in the non-weight-bearing region of the joint, which may lead to donor site disease (Chen et al., 2018). hNCs are a clinically valuable source of cartilage tissue regeneration. hNCs are relatively easier to obtain through a marginally invasive collection program during septal surgery for nasal obstruction, with a lower incidence than chondrocytes obtained from articular cartilage (Lim et al., 2020).

#### 2.9.2 Stem cells

Due to the insuperable limitations of chondrocytes in the treatment of damaged cartilage, a significant amount of research has focused on the research of stem cells in recent years. Stem cells are self-renewing cells that, due to their undifferentiated biology, can produce more stem cells through mitosis or can differentiate into specialized cells (Ma et al., 2018; Yin and Cao, 2021). Various types of stem cells such as ESCs, CSPCs, MSCs, and iPSCs are used to treat cartilage defects (Deng et al., 2020; Johnstone et al., 2020).

#### 2.9.3 Embryonic stem cells

ESCs are derived from inner cell masses of blastocyst embryos and are essentially pluripotent stem cells with the ability to differentiate into all cell types in the body, potentially providing an unlimited supply of cells for cell and tissue therapy and replacement (Toh et al., 2011). However, using ESCs is linked with ethical issues, as induction of ESCs destroys embryos. In addition, ESCs will form teratoma. Because of safety concerns, it is inappropriate to use ESCs for cartilage tissue engineering at this time (Im and Shin, 2015).

**CSPCs:** Articular cartilage has a single cell type, chondrocytes. Although lacking intrinsic repairability, articular cartilage has been proved to contain a population of stem or progenitor cells, similar to those discovered in many other tissues, thought to be relevant to maintaining tissue homeostasis (Jiang et al., 2016). These CSPCs have been found in human, bovine, and horse articular cartilage (Jiang and Tuan, 2015). Li et al. verified the injectable hydrogels based on THA and HB-PEG multi-acrylate macromer containing CSPCs. The secretion of extracellular chondrocyte ECM was enhanced under chondrogenic conditions, and inflammatory gene expression was down-regulated (Li et al., 2020b).

MSCs are undifferentiated pluripotent stem cells characterized by the ability to self-renew when exposed to specific growth signals (Mohamed-Ahmed et al., 2018; Gonzalez-Fernandez et al., 2022). MSCs could differentiate into chondrocytes, osteoblasts, muscle cells, and adipose cells, providing great potential for cartilage tissue engineering. They can be collected from tissues such as bone marrow, umbilical cord blood, adipose, amniotic fluid, pulp, synovium, and even breast milk (Deng et al., 2014; Shao et al., 2015; Mohamed-Ahmed et al., 2018; Fu et al., 2022). MSCs also have immune-enhancing and immunosuppressive effects on the deficiency of primary histocompatibility class II antigens and the secretion of helper T cell type 2 cytokines (Ding et al., 2021).

In particular, BMSCs are considered important seed cells in the treatment of cartilage injury due to their advantages of extensive sources, easy access, strong proliferation ability, significant multidirectional differentiation potential, and the ability to regulate inflammation (Muscolino et al., 2021; Zhu et al., 2022a). Ji et al. studied a temperature-sensitive GM-HPCH injectable hydrogel loaded with BMSCs and TGF-β1. Composite hydrogels can promote the migration of BMSCs, increase the expression of migrated genes, promote the differentiation of BMSCs cartilage, and effectively repair cartilage (Ji et al., 2020). However, ADSCs showed lower levels of immunogenicity than BMSCs. ADSCs showed better stability in the treatment of osteoarthritis. This finding was supported by single-cell analysis results, which clearly showed that ADSCs were more conspecific than BMSCs (Wu et al., 2013; Mazini et al., 2019). Compared with *in vitro* culture conditions, the cell microenvironment *in vivo* can be relatively deficient in oxygen and nutrition. The failure of transplanted cells to adapt to environmental changes may be one of the reasons for the low survival rate of MSCs. Under serum deprivation and hypoxia, ADSCs were more resistant to apoptosis, implying that they may be better adapted to post-transplant conditions (Xu et al., 2017a; Zhou et al., 2019). Boyer et al. and Dehghan-Baniani et al. studied injectable hydrogels loaded with ADSCs *in vivo* and *in vitro*, promoting cartilage defect repair (Boyer et al., 2020; Dehghan-Baniani et al., 2020).

hUCMSCs are also an alternative stem cell source for cartilage tissue engineering. Compared with BMSCs, regarded as standard stem cell sources, which produced more intense type II collagen staining, the hUCMSCs produced more type I collagen and aggregative proteoglycans (Talaat et al., 2020).

**iPSCs:** IPSCs refer to the reprogramming of somatic cells with the potential to be self-renewing and pluripotent stem cells, similar to ESCs, but without the ethical issues and immune response that plague ESCs (Tsumaki et al., 2015; Castro-Viñuelas et al., 2018). In contrast to MSCs' limited differentiation ability after the fourth generation, IPSCs can provide abundant unlimited cell sources with low tumorigenicity (Chang et al., 2020). The potential of IPSCs to differentiate into chondrocytes and its application in cartilage defect modeling have been successfully demonstrated in several researches (Zhang et al., 2020c; Csobonyeiova et al., 2021). He et al. (He et al., 2016) successfully cultured mouse IPSCs to differentiate into chondrocytes based on the sodium alginate hydrogel platform. Xu et al. (Xu et al., 2017b) inoculated human IPSCs with PLCG hydrogel scaffolds and showed repaired chondroid tissues in rabbit cartilage defect models without teratoma.

### 2.10 Stimulating factor

Stimulating factors play an important role in regulating cell proliferation, migration, and differentiation (Zhong et al., 2016; Fan et al., 2020). Many studies have revealed that many cytokines are generally involved in the chondrogenic differentiation of stem cells and maintenance of chondrocyte phenotypes, IGF-1, TGF-β1 or TGF-β3, BMP-2, BMP-4 or BMP-7, and GDF-5 (Campos et al., 2019). Moreover, through genetic engineering to enhance the expression of biologically active molecules, gene therapy offers an alternative method for locally delivering the appropriate stimulus (Huang et al., 2018). Due to the fast clearance of drugs in the joint, much traditional cartilage repair drug therapy has limited efficacy. Injectable hydrogels can maintain drug release and prolong drug retention in the articular cavity. Many studies have been done on injectable hydrogels loaded with drugs (Li et al., 2019).

#### 2.10.1 Transforming growth factor-β

The TGF-β family plays a crucial role in homeostasis and the development of various tissues. Signaling in this protein family mainly activates SMAD-dependent transcription and signaling and SMAD-independent signaling through MAPK such as TAK1 and ERK (Thielen et al., 2019). They regulate cell proliferation, migration, differentiation, and apoptosis and control the degradation and synthesis of ECM. In mammals, there are three isotypes of TGF-β. TGF-β is an inactive soluble protein complex composed of TGF-β dimer, latent TGF-β binding protein, and pre-peptide latency (Blaney Davidson et al., 2007). TGF-β1 is abundant in natural cartilage and controls cartilage ECM production by affecting the synthesis of fibronectin, proteoglycan, and collagen (Zheng et al., 2022). Zhang et al. 2(2021b) developed an injectable hydrogel system based on cross-linked thiolated chitosan and carboxymethyl cellulose as carriers for TGF-β1 in cartilage tissue engineering applications. At 8 weeks postoperatively, hydrogels loaded with TGF-β1 showed excellent repairability in a rat model of full-thickness cartilage defects of the knee. TGF-β3 is also shown to have chondrogenic properties (Martin et al., 2021). Lin et al. 2(2021b) demonstrated *in vitro* that injectable hydrogels loaded with TGF-β3 promoted the expression of chondrogenic genes (Col-2α and ACAN) and decreased the expression of osteogenic genes (Col-1α) in chondrocytes.

#### 2.10.2 Bone morphogenetic protein

BMPs are protein molecules secreted by varieties of cells and are members of TGF-β superfamily. BMP plays a vital role in cartilage and bone formation and is named after its ability to induce cartilage and bone (Deng et al., 2018). BMP promotes SOX9 expression in chondrogenic MSCs. BMP acts upstream of SOX9, and SOX9 is critical for BMP-induced chondrogenesis. SOX9 and BMP participate in a positive feedback loop (Pogue and Lyons, 2006).

#### 2.10.3 Growth/differentiation factor-5

GDF-5 is also a member of TGFβ superfamily. It is a large precursor protein consisting of two main domains: the active C-terminal domain and the N-terminal precursor domain with signal sequences and cleavage sites. GDF-5 overexpression can promote chondrogenesis, which improves MSC adhesion and chondrocyte proliferation (Sun et al., 2021). However, GDF-5 promotes osteogenesis and hypertrophy, limiting its therapeutic effect on cartilage repair. Therefore, it is better to control the anabolism and anti-catabolism of GDF-5 on chondrocytes and apply it to cartilage tissue engineering (Mang et al., 2020).

#### 2.10.4 Insulin-like growth factor-1

IGF-1 is an anabolic growth factor that promotes cell proliferation and inhibits apoptosis and is vital in chondrogenesis and homeostasis. IGF-1 is a crucial factor promoting cartilage matrix anabolism in synovial fluid and serum. In addition to stimulating ECM production, IGF-1 can stimulate MSCs proliferation and chondrogenic differentiation (Wen et al., 2021). Many studies have demonstrated the effectiveness of IGF-1 in articular cartilage repair, and it is dose-dependent (Wei et al., 2020). High-dose IGF-1 is more conducive to the formation and integration of cartilage regeneration, while low-dose IGF-1 is more conducive to subchondral bone (Zhang et al., 2017).

#### 2.10.5 Platelet-rich plasma

PRP is rich in various growth factors, cytokines, and proteins, and many studies have demonstrated the potential effectiveness and excellent biocompatibility of PRP for cartilage defect repair (Yan et al., 2020). PL is a natural GFs pool consisting of TGF-β1, TGF-β3, IGF-1, and VEGF and can be prepared by simple PRP thermal cycling. Due to the removal of platelet fragments by gradient centrifugation, the immunogenicity of PL is lower than that of PRP (Li et al., 2021b). Tang and his team encapsulated PL using EPL and heparin NPs into injectable hydrogels. The injectable hydrogel ameliorated early cartilage degeneration and promoted late cartilage repair in rats with knee arthritis (Tang et al., 2021).

#### 2.10.6 Kartogenin

KGN is a stable, nonprotein small molecule with a structure of 2- [(4-phenyl) carbamoyl] benzoic acid that induces the differentiation of BMSCs into chondrocytes regulating the CBFβ-RUNX1 signaling pathway (Yuan et al., 2021b). It is more effective than growth factors in inducing cartilage regeneration and has been processed and applied in various forms in cartilage tissue engineering (Cai et al., 2019). Dehghan Baniani et al. (Dehghan-Baniani et al., 2020) incorporated KGN into a thermosensitive injectable chitosan hydrogel. KGN can be released continuously for more than 40 days and promote chondrogenic differentiation of human ADSCs *in vitro* (including upregulation of COL2A, SOX9, and ACAN chondrogenic genes).

#### 2.10.7 Gene therapy

It refers to delivering nucleic acids to tissues of interest by direct (*in vivo*) or transduced cell-mediated (*in vitro*) methods using viral and non-viral vectors. In the past few decades, the strategy of expressing therapeutic transgenes at injured sites has been adopted to promote cartilage repair (Grol and Lee, 2018). However, the problems associated with non-standard procedures remain unresolved. In addition, the association of gene therapy with tissue engineering may be a promising strategy for treating cartilage and osteochondral damage (Bellavia et al., 2018). Several clinical trials of gene therapy have been conducted in patients with end-stage knee OA by intraarticular injection of human adolescent chondrocytes overexpressing cDNA encoding TGF-β1 with retroviral vectors. In the latest placebo-controlled randomized trial, clinical scores improved in the gene therapy group compared with placebo (Madry and Cucchiarini, 2016). Zhu et al. (2022b) used an injectable hydrogel to deliver miR-29b-5p (aging-associated miRNA), which was functionalized by binding to stem cell homing peptide SKPPGTSS for SMSCs recruitment contemporaneously. Sustained miR-29b-5p transport and recruitment of SMSCs, followed by chondrocyte differentiation, results in successful chondrocyte regeneration and cartilage repair. Yu et al. (2021b) implanted genetically modified ADSCs overexpressing TGF-β1 into injectable ECM hydrogels. In the rat OA model, intraarticular injection of hydrogels loaded with ADSCs overexpressing TGF-β1 significantly reduced joint inflammation, cartilage degeneration, and subchondral bone loss.

#### 2.10.8 Drug

Until now, the conventional treatment for OA has been to reduce the main symptoms with oral or topical injections of various drugs, including NSAIDs, analgesics, and corticosteroids. The efficacy of local injection is hampered by their rapid diffusion, instability, and low retention at the target site (Mok et al., 2020). More importantly, frequent oral use of these drugs can cause serious side effects, such as increased throw of cardiovascular disease and stimulus of the gastrointestinal tract (García-Couce et al., 2022). Suitable injectable hydrogel delivery systems could sustainably release therapeutic drug concentrations in cartilage (Cao et al., 2021a; Cao et al., 2021b; Shi et al., 2021b; Khan et al., 2022). Hanafy and El-Ganainy, 2020 prepared an injectable hydrogel based on Poloxamer 407 and HA loaded with the anti-inflammatory drug DK. 40% of DK was released after 4 days. Injectable hydrogels maintain DK content and drug release percentage after 3 months of storage. Loaded DK injectable hydrogel had the greatest anti-edematous and anti-nociceptive effect compared to oral and direct injection DK. Both histomorphology and radiology showed regeneration of cartilage defects. Branco et al. (2022) prepared an injectable PVA-based hydrogel that continuously released diclofenac for cartilage regeneration.

Some of the following drugs are also used in injectable hydrogels to repair cartilage damage. Dexamethasone, a glucocorticoid, has 20–30 times the anti-inflammatory potency of natural hydrocortisone. It can reduce the loss of collagen and proteoglycan in ECM, maintain ECM synthesis, and maintain the viability of chondrocytes. In addition, it is a key reagent for inducing chondrogenesis of MSCs *in vitro* (García-Fernández et al., 2020; Wang et al., 2021e; García-Couce et al., 2022). GlcN is a naturally occurring amino monosaccharide, widely used to reduce joint pain and repair cartilage (Suo et al., 2020; Zhang et al., 2021c). Quercetin and naringin are flavonoids widely found in fruits and vegetables with strong anti-inflammatory and antioxidant effects. It can inhibit ECM degradation, reduce the inflammatory response and maintain chondrocyte phenotype (Yu et al., 2020b; Li et al., 2021d). Icariin can improve cartilage ECM synthesis and restrain ECM degradation, up-regulate chondrogenic specific gene expression of chondrocytes, and induce oriented chondrogenesis of BMSCs without hypertrophic differentiation (Zhu et al., 2022a).

## 3 Advanced injectable hydrogels for cartilage repair tissue engineering

In recent years, various injectable hydrogels with good plasticity and biological properties have been widely studied for cartilage repair tissue engineering (Table 1). Many studies have investigated the regeneration potential of injectable hydrogel cartilage *in vitro* and *in vivo*. Fattahpour et al. (2020) developed and characterized MC-CMC-Pluronic and ZnCl_2_ injectable hydrogels containing meloxicam. The release time of meloxicam in hydrogels containing nanoparticles was significantly longer than in hydrogels without nanoparticles. This injectable hydrogel showed good chondrocyte adhesion and proliferation. Qi et al. (2018) prepared a photo-crosslinked injectable SerMA hydrogel loaded with chondrocytes. After 8 weeks of implantation, SerMA hydrogel loaded with chondrocytes successfully formed regenerative cartilage in rabbits. Most importantly, regenerated cartilage is structurally similar to natural cartilage (Qi et al., 2018, Qi et al., 2021).

**TABLE 1:** Application of some advanced injectable hydrogels in cartilage tissue engineering.

Model	Formation	Technique	Structure	Major materials	Cell	Stimulating factor	Ref
Minipig	Chemical crosslinking	Photopolymerization	Nanocomposite hydrogel	HA/ PLGA	—	KGN	Cao et al. (2021a)
Rat	Physical crosslinking	Thermosensitive	Dual networks	Alginate/ Bioglass	—	Quercetin	Wen et al. (2021)
Rabbit	Chemical crosslinking	Schiff base chemistry/Photopolymerization	Dual networks	Alginate/Amino gelatin<	—	TGF-β3/KGN	Deng et al. (2014)
Rat	Chemical crosslinking	Enzyme-mediated crosslinking	Dual networks	Col I/ tyramine hyaluronic acid	BMSCs	TGF-β1	Tang et al. (2021)
*In vitro*	Physical crosslinking	—	—	Amnion membrane	ADSCs (rat)	—	Kim et al. (2017)
*In vitro*	Physical crosslinking	Ionic interaction	Nanocomposite hydrogel	Carboxymethyl chitosan/ methylcellulose/ Pluronic F127/ ZnCl_2_	Chondrocytes (sheep)	Meloxicam	Jiang et al. (2021)
Mice	Chemical crosslinking	Enzyme-mediated crosslinking	Dual networks	HA/ gelatin/ EGCG	—	—	Liu and Lin, (2019)
Rabbit	Physical crosslinking	Thermosensitive	Microspheres hydrogel	Pluronic F127/ PLGA	BMSCs	BMP-2	Yan et al. (2020)
Rat	Chemical crosslinking	Photopolymerization	particle scaffolding hydrogel	PEG-MAL/ PEG thiol/ arginine-glycine-aspartic acid cell adhesive peptide/ CS	—	—	Fu et al. (2018)
Rabbit	Chemical crosslinking	Schiff base chemistry	Semi-IPN	Gelatin/ HA/ Dex-ox	—	Naproxen/ Dexamethasone	Wang et al. (2020b)
Canine	—	—	—	Silanised hydroxypropymethyl cellulose/ silanised chitosan	ADSCs	-	Johnstone et al. (2020)
*In vitro*	Chemical crosslinking	Enzyme-mediated crosslinking	Dual networks	Collagen/ gelatin/ hydroxy-phenyl-propionic acid	Chondrocytes (bovine)	-	Monsef and Salavati-Niasari, (2021)
*In vitro*	Chemical crosslinking	Click chemistry	Nanocomposite hydrogel	PEGDGE/ PAMAM/ silica nanoparticles/ silver nanoparticles	—	Isoniazid/ rifampicin	De France et al. (2016)
Human *ex vivo* / rabbit	Chemical crosslinking	Photopolymerization	Traditional	GelMA/ FITC fluorophore	ADSCs/ BMSCs	-	García-Couce et al. (2022)
Rat	Physical/chemical crosslinking	Thermosensitive/photopolymerization	Traditional	Hydroxypropyl chitin/ methacrylate	BMSCs	TGF-β1	Chen et al. (2018)
*In vitro*	Physical/chemical crosslinking	Thermosensitive	Microspheres hydrogel	methoxy poly (ethylene glycol)-poly (alanine)/ PLGA	Chondrocytes (rat)	TGF-β3	Han et al. (2021)
Rat	Physical crosslinking	Ionic interaction	Microspheres hydrogel	sodium alginate / bioglass/ δ-Gluconolactone	—	Strontium	Lee and Mooney, (2012)
Goat	Chemical crosslinking	Enzyme-mediated crosslinking	Dual networks	Silk fibroin/ CMC/ gelatin	ADSCs	—	Cai et al. (2019)
Rat	Physical crosslinking	Thermosensitive	Nanocomposite hydrogel	PLEL/ EPL	—	Platelet lysate	Castro-Viñuelas et al. (2018)
Rat	Chemical crosslinking	Disulfide crosslinking	Dual networks	Thiolated chitosan/ carboxy-methyl cellulose	—	TGF-β1	Echave et al. (2019)
Rat	—	—	Microspheres hydrogel	PLGA/ chitosan/ gelatin	—	Platelet lysate	Gomez-Florit et al. (2020)
Rat	Chemical crosslinking	Photopolymerization	Traditional	Sericin/ methacrylogy groups	Chondrocytes	—	Yuan et al. (2021b)
*In vitro*	Chemical crosslinking	Enzyme-mediated crosslinking	IPN	Alginate/ cartilage silk fibroin extracellular matrix/	Chondrocytes (human)	—	Song et al. (2015)
Rat	Chemical crosslinking	Photopolymerization	Microspheres hydrogel	GelMA	—	Diclofenac sodium	Zinatloo-Ajabshir et al. (2018)
Rat	Chemical crosslinking	Photopolymerization	IPN	GelMA	—	—	Nicol, (2021)
Rabbit	-	-	-	DNA	BMSCs	—	Graça et al. (2020)
Rabbit	Chemical crosslinking	Schiff base chemistry	Dual networks	Chitosan/ HA	ADSCs	Chondrocyte EVs	Mok et al. (2020)
Rat	Chemical crosslinking	Silanization	IPN	Chitosan/ HA/ silanized-hydroxypropyl methylcellulose	Chondrocytes	-	Li et al. (2021c)
Rat	Chemical crosslinking	Enzyme-mediated crosslinking	Double networks	Alginate/ dopamine/ CS/ silk fibroin	—	BMSCs EVs	Zhu et al. (2022b)
*In vitro*	Chemical crosslinking	Chemical crosslinking	Microspheres hydrogel	PLGA/ carboxymethyl chitosan-oxidized chondroitin sulfate	BMSCs (rabbit)	KGN	Eyre, (2004)
*In vitro*	Physical crosslinking	Thermosensitive	Microspheres hydrogel	Chitosan/ human acellular cartilage ECM	BMSCs (human)	-	Mehrali et al. (2017)
Rat	Physical crosslinking	Thermosensitive	Dual networks	PDLLA-PEG-PDLLA	-	SMSCs EVs/ circRNA3503	Yu et al. (2021b)
*In vitro*	Chemical crosslinking	Click chemistry	Dual networks	PEG/ CS	ADSCs (rat)	—	Zhang et al. (2021b)
Rabbit	Physical crosslinking	Guest-host complexation	Microspheres hydrogel	HA–cyclodextrin/ polyacrylic acid–ferrocene/ PLGA	Chondrocytes	GSH/ iron oxide nanoparticles	Qi et al. (2018)
Pig explants	Physical crosslinking	Thermosensitive	Dual networks	PLL/ poly (N-isopropylacrylamide	Chondrocytes/ MSCs (rabbit)	—	Saravanan et al. (2019)
Rat	Physical crosslinking	Thermosensitive	Dual networks	Sodium alginate/ bioglass	—	Naringin	Wei et al. (2020)
*In vitro*	Physical/chemical crosslinking	Ionic interaction	IPN	GelMA/ HA	—	—	Rinoldi et al. (2021)
Rat	Physical crosslinking	Thermosensitive	Nanoparticle hydrogel	Poly organosphosphazenes	—	TCA	Lokhande et al. (2018)
Rat	Physical crosslinking	pH-responsive	IPN	Thiolated HA/ Col I	Gene-engineered ADSCs overexpressing TGF-β1	—	Fan et al. (2020)
*In vitro*	Physical crosslinking	Thermosensitive	Traditional	Chitosan/ N-(β- maleimidopropyloxy) succinimide ester/ β-glycerophosphate	ADSCs (human)	KGN	Deng et al. (2020)
Rat	Chemical crosslinking	Dynamic chemical bonds	Dual networks	Glycol chitosan/ GCS/DF-PEG	ADSCs	—	Kahn et al. (2017)
*In vitro*	Chemical crosslinking	Ionic interaction	Microspheres hydrogel	Decellularized bovine articular Cartilage/ alginate	BMSCs (human)	—	Werkmeister et al. (2010)
Rat	Chemical crosslinking	—	—	SAP	—	miR-29b-5p	Xu et al. (2017b)
Rat	Physical crosslinking	Thermosensitive	IPN	HA/ Poloxamer 407	BMSCs	Icariin	Cai et al. (2020)
*In vitro*	Chemical crosslinking	Photopolymerization	Double networks	GelMA/ HA/ hyaluronic acid methacrylate	Chondrocytes (rabbit)	—	Meng et al. (2019)
Rat	Physical crosslinking	Thermosensitive	Nanoparticle hydrogel	Chitosan/ silk fibroin/ glycerophosphate	BMSCs	TGF-β1	Luu et al. (2022)

Numerous studies have investigated injectable hydrogel strategies that guide stem cell phenotypic expression and manipulate cartilage matrix properties. Liu et al. (2021b) revealed that hydrogel scaffolds with gradient distribution could better simulate the function of natural cartilage and promote stem cell differentiation than homogeneous hydrogel scaffolds. In rabbit models, injectable hydrogels containing BMSCs that sustained-release BMP-2 were more effective than microfractures alone in treating cartilage damage (Vayas et al., 2021). Zhang and his team prepared injectable hydrogels composed of hyaluronic acid-tyramine and collagen type I-tyramine-loaded BMSCs and TGF-β1 (Zhang et al., 2020d). The injectable hydrogel supports the differentiation of BMSCs into chondrocytes. *In vivo* experiments further demonstrated that this injectable hydrogel can achieve good repair of transparent articular cartilage. Mahajan et al. (2022) developed a silk fibroin/CMC/gelatin complex hydrogel that increases contraction and hardness over time. The contractile-mediated mechanical stimulation promotes the formation of ADSCs cartilage. The regenerated cartilage of goats is very similar to natural cartilage. The cells may have a therapeutic effect because EVs derived from them can induce stem cell differentiation and chondrocyte proliferation (Liu et al., 2017b; Saveh-Shemshaki et al., 2019; Song et al., 2021). Zhang et al. (Zhang et al., 2021d) prepared the load with BMSCs-EVs alginate/dopamine/CS/silk fibroin composite injectable hydrogel.

When the hydrogel was injected into a rat cartilage defect model, EVs released by injectable hydrogel could recruit BMSCs into the hydrogel through a chemokine signaling pathway and promote BMSCs proliferation and differentiation to promote cartilage repair. Tao et al. (2021) implanted circRNA3503 carried by SMSCS-EVS into PDLLA-PEG-PDLLA injectable hydrogel. EVs promoted chondrocyte migration and proliferation, while circRNA3503 reduced chondrocyte apoptosis and ECM degradation, and both of them combined with regenerating damaged cartilage in rats. Heirani-Tabasi et al. (2021) demonstrated that human articular chondrocytes EVs in chitosan-HA injectable hydrogels have chondrogenic differentiation effects on ADSCs. In the rabbit cartilage defect model, EVS-treated ADSCs had greater cartilage regeneration ability than untreated MSCs or ADSCs treated with EVS without gel.

In addition, injectable hydrogels have been studied to detect cartilage repair ability dynamically. According to Onofrillo et al. (Onofrillo et al., 2021), FLIH was considered a sensitive tool for monitoring the photo-crosslinked injectable hydrogels in cartilage tissue engineering structure. The generation of cartilage ECM in injectable hydrogels is related to the fluorescence loss curve, which describes the hydrogels’ degradation rate. Using FLIH can be achieved through an extensible system for sample maintenance and fluorescence recording, resulting in an analytical real-time monitoring system suitable for non-contact high-throughput evaluation of chondrogenesis.

## 4 Summary and perspectives

The repair of cartilage defects still faces many challenges. Injectable hydrogel is the main development direction of cartilage tissue engineering, not only because of its bionic properties similar to cartilage ECM due to its high moisture content but also because of its minimally invasive properties and strong plasticity ability to match irregular defects. First, to improve the biomechanical properties of injectable hydrogels, traditional single-network hydrogels are added with different polymer mixtures or networks, and many nano/micron-composite materials are used to alter the mechanical properties and sustained-release properties of the matrix. Integrating cells and cytokines or other stimulators into injectable hydrogels can improve the integration of hydrogels with surrounding cartilage and promote cartilage regeneration. Controlling the proliferation and differentiation of stem cells into chondrocytes is of great interest.

Despite many relatively successful preclinical studies and several advanced manufacturing methods for engineered tissues, there remain limitations that must be addressed in preparing injectable hydrogels with excellent performance for optimal regeneration of cartilage defects. First, the injectable hydrogel matrix must be able to fill the defect area with a smooth surface similar to natural cartilage without fusing with the surrounding healthy tissue. Second, rapid degradation of the hydrogel matrix before replacement by regenerative ECM may compromise its mechanical stability and therapeutic efficacy. To address this issue, appropriate exogenous cells (such as MSCs) can be added to the hydrogel matrix, or peripheral cartilage cells can be recruited to the defect area, where they generate new cartilage tissue to replace the degraded hydrogel smatrix. Therefore, the signaling pathway from stem cells to specific chondrocytes and the specific stimulation mechanism in hydrogel must be further understood. Finally, injectable hydrogels need to be further studied at the clinical level, from experimental animals to human experiments, and thoroughly evaluated factors such as biocompatibility, degradability, and comfort of hydrogel materials.

## Glossary

ECM: Extracellular matrix
OA: Osteoarthritis
NSAIDs: Non-steroidal anti-inflammatory drugs
ACI: Autologous chondrocyte implantation
HRP: Horseradish peroxidase
H2O2: Hydrogen peroxide
CST: Critical solution temperature
NIPAAm: N-isopropyl acrylamide
IPN: Interpenetrating Polymer Network
IUPAC: International Union of Pure and Applied Chemistry
GE: Genipin
HA-SH: Thiolated hyaluronic acid
N/MPs: Nano/micron particles
NPs: Nanoparticles
CNCs: Cellulose nanocrystals
POEGMA: Poly (oligoethylene glycol methacrylate)
MW: Molecular weight;
RGD: Rrginine-glycine-aspartate
PLGA: Poly lactic-co-glycolic acid
PLA: Polylactic acid
PGA: Polyglycolic acid
PCL: Polycaprolactone
PVA: Polyvinyl alcohol
PLL: Poly l‐lysine
MPA: Methoxy poly (ethylene glycol)-poly (alanine)
PL: Platelet lysates
GFs: Growth factors
IGF-1: Insulin-like growth factor
VEGF: Vascular endothelial growth factor
TGF-β: Transforming growth factor-β
TGF-β1: Transforming growth factor-β1
TGF-β3: Transforming growth factor-β3
BMP-2: Bone morphogenetic protein-2
BMP-4: Bone morphogenetic protein-4
BMP-7: Bone morphogenetic protein-7
GDF-5: Growth/differentiation factor-5
PRP: Platelet-rich plasma
PDLLA: Poly (D, l-lactide)
PEG: Polyethylene glycol
HB-PEG: Hyperbranched PEG
PEGDA: PEG diacrylate
PEG-MAL: PEG maleimide
GCS: Glycol chitosan
DF-PEG: Dibenzaldehyde-terminated PEG
PAMAM: Polyamidoamine dendrimer
EGCG: Epigallocatechin-3-gallate
CS: Chondroitin sulfate
HA: Hyaluronic acid
GAG: Glycosaminoglycans
GelMA: Gelatin methacrylate
HAMA: Methacrylated hyaluronic acid
PBA: 3-Aminophenylboronic acid
EPL: ε-poly-l-lysine
PLEL: Poly (d, l-lactide)-poly (ethylene glycol)-poly (d, l-lactide)
DNA: Deoxyribonucleic acid
EVs: Extracellular vesicles
MSCs: Mesenchymal stem cells
ADSCs: Adipose mesenchymal stem cells
BMSCs: Bone marrow mesenchymal stem cells
SMSCs: Synovium mesenchymal stem cells
CSPCs: Chondrogenic stem/progenitor cells
THA: Thiolfunctionalised hyaluronic acid
KGN: Kartogenin
circRNA3503: Circular RNA 3503
GSH: Glutathione
Dex-ox: Oxidized dextran
FITC: Fluorescein isothiocyanate
CMC: Carboxymethyl cellulose
SF: Silk fibroin
TCA: Triamcinolone acetonide
Col I: Type I collagen
Col 1α: Type 1α collagen
Col 2α: Type 2α collagen
miR-29b-5p: MicroRNA-29b-5p
SAP: Serum amyloid P component
ROS: Reactive oxygen species
MMP13: Matrix metallopeptidase13
3D: Third Dimension
COL2: Type II collagen
ACAN: Aggrecan
hNCs: Human nasal septal cartilage
hUCMSCs: Human umbilical cord mesenchymal stem cells
CBFβ-RUNX1: Core-binding factor β-Runt-related transcription factor 1
SKPPGTSS: A stem cell-homing peptide
DK: Diclofenac potassium
GlcN: Glucosamine
MC: Methylcellulose
MC-CMC: Methylcellulose-carboxymethyl chitosan
FLIH: Fluorescently labeled injectable hydrogels
Si-HPMC: Silanized-hydroxypropyl methylcellulose
HA–pAA: Hyaluronic acid–polyacrylic acid
iPSCs: Induced Pluripotent Stem Cells
ESCs: Embryonic stem cells
GM-HPCH: Glycidyl methacrylate-modified-hydroxypropyl chitin hydrogel
PLCG: Poly(lactic-co-glycolide)
SMAD: Small mothers against decapentaplegic
TAK1: Transforming growth factor beta-activated kinase1
ERK: Extracellular signal-regulated kinase
SOX9: SRY-related high-mobility-group box 9
SerMA: Sericin methacryloyl

## References

[B1] Abasalizadeh F. Moghaddam S. V. Alizadeh E. akbari E. Kashani E. Fazljou S. M. B. (2020). Alginate-based hydrogels as drug delivery vehicles in cancer treatment and their applications in wound dressing and 3D bioprinting. J. Biol. Eng. 14, 8. 10.1186/s13036-020-0227-7 32190110 PMC7069202

[B2] Abdulghani S. Morouço P. G. (2019). Biofabrication for osteochondral tissue regeneration: Bioink printability requirements. J. Mat. Sci. Mat. Med. 30 (2), 20. 10.1007/s10856-019-6218-x 30689057

[B3] Ahmadian-Fard-Fini S. Ghanbari D. Amiri O. Salavati-Niasari M. (2020). Electro-spinning of cellulose acetate nanofibers/Fe/carbon dot as photoluminescence sensor for mercury (II) and lead (II) ions. Carbohydr. Polym. 229, 115428. 10.1016/j.carbpol.2019.115428 31826498

[B4] Ali F. Khan I. Chen J. Akhtar K. Bakhsh E. M. Khan S. B. (2022). Emerging fabrication strategies of hydrogels and its applications. Gels 8 (4), 205. 10.3390/gels8040205 35448106 PMC9024659

[B5] Aminabhavi T. M. Nadagouda M. N. More U. A. Joshi S. D. Kulkarni V. H. Noolvi M. N. (2015). Controlled release of therapeutics using interpenetrating polymeric networks. Expert Opin. Drug Deliv. 12 (4), 669–688. 10.1517/17425247.2014.974871 25341410

[B6] Amiri M. Salavati-Niasari M. Pardakhty A. Ahmadi M. Akbari A. (2017). Caffeine: A novel green precursor for synthesis of magnetic CoFe2O4 nanoparticles and pH-sensitive magnetic alginate beads for drug delivery. Mat. Sci. Eng. C Mat. Biol. Appl. 76, 1085–1093. 10.1016/j.msec.2017.03.208 28482472

[B7] Antoniuk I. Amiel C. (2016). Cyclodextrin-mediated hierarchical self-assembly and its potential in drug delivery applications. J. Pharm. Sci. 105 (9), 2570–2588. 10.1016/j.xphs.2016.05.010 27342436

[B8] Arkenberg M. R. Nguyen H. D. Lin C-C. (2020). Recent advances in bio-orthogonal and dynamic crosslinking of biomimetic hydrogels. J. Mat. Chem. B 8 (35), 7835–7855. 10.1039/d0tb01429j PMC7574327 32692329

[B9] Armiento A. R. Alini M. Stoddart M. J. (2019). Articular fibrocartilage - why does hyaline cartilage fail to repair? Adv. Drug Deliv. Rev. 146, 289–305. 10.1016/j.addr.2018.12.015 30605736

[B10] Armiento A. R. Stoddart M. J. Alini M. Eglin D. (2018). Biomaterials for articular cartilage tissue engineering: Learning from biology. Acta Biomater. 65, 1–20. 10.1016/j.actbio.2017.11.021 29128537

[B11] Asadi N. Alizadeh E. Salehi R. Khalandi B. Davaran S. Akbarzadeh A. (2018). Nanocomposite hydrogels for cartilage tissue engineering: A review. Artif. Cells Nanomed. Biotechnol. 46 (3), 465–471. 10.1080/21691401.2017.1345924 28743188

[B12] Balakrishnan B. Joshi N. Jayakrishnan A. Banerjee R. (2014). Self-crosslinked oxidized alginate/gelatin hydrogel as injectable, adhesive biomimetic scaffolds for cartilage regeneration. Acta Biomater. 10 (8), 3650–3663. 10.1016/j.actbio.2014.04.031 24811827

[B13] Behan K. Dufour A. Garcia O. Kelly D. (2022). Methacrylated cartilage ECM-based hydrogels as injectables and bioinks for cartilage tissue engineering. Biomolecules 12 (2), 216. 10.3390/biom12020216 35204718 PMC8961582

[B14] Bellavia D. Veronesi F. Carina V. Costa V. Raimondi L. De Luca A. (2018). Gene therapy for chondral and osteochondral regeneration: Is the future now? Cell. Mol. Life Sci. 75 (4), 649–667. 10.1007/s00018-017-2637-3 28864934 PMC11105387

[B15] Bhattacharjee M. Escobar Ivirico J. L. Kan H-M. Shah S. Otsuka T. Bordett R. (2022). Injectable amnion hydrogel-mediated delivery of adipose-derived stem cells for osteoarthritis treatment. Proc. Natl. Acad. Sci. U. S. A. 119 (4), e2120968119. 10.1073/pnas.2120968119 35046053 PMC8794776

[B16] Bhattacharjee M. Ivirico J. L. E. Kan H-M. Bordett R. Pandey R. Otsuka T. (2020). Preparation and characterization of amnion hydrogel and its synergistic effect with adipose derived stem cells towards IL1β activated chondrocytes. Sci. Rep. 10 (1), 18751. 10.1038/s41598-020-75921-w 33127964 PMC7603317

[B17] Bian J. Cai F. Chen H. Tang Z. Xi K. Tang J. (2021). Modulation of local overactive inflammation via injectable hydrogel microspheres. Nano Lett. 21 (6), 2690–2698. 10.1021/acs.nanolett.0c04713 33543616

[B18] Blaney Davidson E. N. van der Kraan P. M. van den Berg W. B. (2007). TGF-beta and osteoarthritis. Osteoarthr. Cartil. 15 (6), 597–604. 10.1016/j.joca.2007.02.005 17391995

[B19] Bordbar S. Lotfi Bakhshaiesh N. Khanmohammadi M. Sayahpour F. A. Alini M. Baghaban Eslaminejad M. (2020). Production and evaluation of decellularized extracellular matrix hydrogel for cartilage regeneration derived from knee cartilage. J. Biomed. Mat. Res. A 108 (4), 938–946. 10.1002/jbm.a.36871 31894891

[B20] Boyer C. Réthoré G. Weiss P. d’Arros C. Lesoeur J. Vinatier C. (2020). A self-setting hydrogel of silylated chitosan and cellulose for the repair of osteochondral defects: From *in vitro* characterization to preclinical evaluation in dogs. Front. Bioeng. Biotechnol. 8, 23. 10.3389/fbioe.2020.00023 32117912 PMC7025592

[B21] Branco A. C. Oliveira A. S. Monteiro I. Nolasco P. Silva D. C. Figueiredo-Pina C. G. (2022). PVA-based hydrogels loaded with diclofenac for cartilage replacement. Gels 8 (3), 143. 10.3390/gels8030143 35323256 PMC8954927

[B22] Bustamante-Torres M. Romero-Fierro D. Arcentales-Vera B. Palomino K. Magana H. Bucio E. (2021). Hydrogels classification according to the physical or chemical interactions and as stimuli-sensitive materials. Gels 7 (4), 182. 10.3390/gels7040182 34842654 PMC8628675

[B23] Cai G. Liu W. He Y. Huang J. Duan L. Xiong J. (2019). Recent advances in kartogenin for cartilage regeneration. J. Drug Target. 27 (1), 28–32. 10.1080/1061186X.2018.1464011 29772932

[B24] Cai H. Wang P. Xu Y. Yao Y. Liu J. Li T. (2020). BMSCs-assisted injectable Col I hydrogel-regenerated cartilage defect by reconstructing superficial and calcified cartilage. Regen. Biomater. 7 (1), 35–45. 10.1093/rb/rbz028 32153990 PMC7053261

[B25] Cai Z. Li Y. Song W. He Y. Li H. Liu X. (2021). Anti-inflammatory and prochondrogenic in situ-formed injectable hydrogel crosslinked by strontium-doped bioglass for cartilage regeneration. ACS Appl. Mat. Interfaces 13 (50), 59772–59786. 10.1021/acsami.1c20565 34898167

[B26] Campos Y. Almirall A. Fuentes G. Bloem H. L. Kaijzel E. L. Cruz L. J. (2019). Tissue engineering: An alternative to repair cartilage. Tissue Eng. Part B Rev. 25 (4), 357–373. 10.1089/ten.TEB.2018.0330 30913997

[B27] Cao J. Su J. An M. Yang Y. Zhang Y. Zuo J. (2021). Correction to "novel DEK-targeting aptamer delivered by a hydrogel microneedle attenuates collagen-induced arthritis. Mol. Pharm. 18 (11), 4231. 10.1021/acs.molpharmaceut.1c00732 34582206

[B28] Cao J. Su J. An M. Yang Y. Zhang Y. Zuo J. (2021). Novel DEK-targeting aptamer delivered by a hydrogel microneedle attenuates collagen-induced arthritis. Mol. Pharm. 18 (1), 305–316. 10.1021/acs.molpharmaceut.0c00954 33253580

[B29] Castro-Viñuelas R. Sanjurjo-Rodríguez C. Piñeiro-Ramil M. Hermida-Gomez T. Fuentes-Boquete I. de Toro-Santos F. (2018). Induced pluripotent stem cells for cartilage repair: Current status and future perspectives. Eur. Cell. Mat. 36, 96–109. 10.22203/eCM.v036a08 30204229

[B30] Chang Y-H. Wu K-C. Ding D-C. (2020). Induced pluripotent stem cell-differentiated chondrocytes repair cartilage defect in a rabbit osteoarthritis model. Stem Cells Int. 2020, 1–16. 10.1155/2020/8867349 PMC7671807 33224204

[B31] Chen J. Yang J. Wang L. Zhang X. Heng B. C. Wang D. A. (2021). Modified hyaluronic acid hydrogels with chemical groups that facilitate adhesion to host tissues enhance cartilage regeneration. Bioact. Mat. 6 (6), 1689–1698. 10.1016/j.bioactmat.2020.11.020 PMC7708943 33313448

[B32] Chen W. Li C. Peng M. Xie B. Zhang L. Tang X. (2018). Autologous nasal chondrocytes delivered by injectable hydrogel for *in vivo* articular cartilage regeneration. Cell Tissue Bank. 19 (1), 35–46. 10.1007/s10561-017-9649-y 28815373 PMC5829115

[B33] Chiang M-Y. Cheng I. Y. Chou S-H. Tsai J. H. Chen Y. J. Lu H. E. (2021). A smart injectable composite hydrogel with magnetic navigation and controlled glutathione release for promoting *in situ* chondrocyte array and self-healing in damaged cartilage tissue. J. Mat. Chem. B 9 (45), 9370–9382. 10.1039/d1tb02030g 34726686

[B34] Cho H. Kim J. Kim S. Jung Y. C. Wang Y. Kang B. J. (2020). Dual delivery of stem cells and insulin-like growth factor-1 in coacervate-embedded composite hydrogels for enhanced cartilage regeneration in osteochondral defects. J. Control. Release 327, 284–295. 10.1016/j.jconrel.2020.08.002 32763434

[B35] Choi J. H. Park A. Lee W. Youn J. Rim M. A. Kim W. (2020). Preparation and characterization of an injectable dexamethasone-cyclodextrin complexes-loaded gellan gum hydrogel for cartilage tissue engineering. J. Control. Release 327, 747–765. 10.1016/j.jconrel.2020.08.049 32941931

[B36] Coenen A. M. J. Bernaerts K. V. Harings J. A. W. Jockenhoevel S. Ghazanfari S. (2018). Elastic materials for tissue engineering applications: Natural, synthetic, and hybrid polymers. Acta Biomater. 79, 60–82. 10.1016/j.actbio.2018.08.027 30165203

[B37] Cross L. M. Thakur A. Jalili N. A. Detamore M. Gaharwar A. K. (2016). Nanoengineered biomaterials for repair and regeneration of orthopedic tissue interfaces. Acta Biomater. 42, 2–17. 10.1016/j.actbio.2016.06.023 27326917

[B38] Csobonyeiova M. Polak S. Nicodemou A. Zamborsky R. Danisovic L. (2021). iPSCs in modeling and therapy of osteoarthritis. Biomedicines 9 (2), 186. 10.3390/biomedicines9020186 33673154 PMC7917981

[B39] Das S. K. Farooqi A. (2008). Osteoarthritis. Best. Pract. Res. Clin. Rheumatol. 22 (4), 657–675. 10.1016/j.berh.2008.07.002 18783743

[B40] Davar F. Salavati-Niasari M. Fereshteh Z. (2010). Synthesis and characterization of SnO2 nanoparticles by thermal decomposition of new inorganic precursor. J. Alloys Compd. 496 (1), 638–643. 10.1016/j.jallcom.2010.02.152

[B41] De France K. J. Chan K. J. W. Cranston E. D. Hoare T. (2016). Enhanced mechanical properties in cellulose nanocrystal-poly(oligoethylene glycol methacrylate) injectable nanocomposite hydrogels through control of physical and chemical cross-linking. Biomacromolecules 17 (2), 649–660. 10.1021/acs.biomac.5b01598 26741744

[B42] Dehghan-Baniani D. Chen Y. Wang D. Bagheri R. Solouk A. Wu H. (2020). Injectable *in situ* forming kartogenin-loaded chitosan hydrogel with tunable rheological properties for cartilage tissue engineering. Colloids Surfaces B Biointerfaces 192, 111059. 10.1016/j.colsurfb.2020.111059 32380404

[B43] Deng S. Huang R. Wang J. Zhang S. Chen Z. Wu S. (2014). Miscellaneous animal models accelerate the application of mesenchymal stem cells for cartilage regeneration. Curr. Stem Cell Res. Ther. 9 (3), 223–233. 10.2174/1574888x09666140213201331 24524791

[B44] Deng Z. Jin J. Wang S. Qi F. Chen X. Liu C. (2020). Narrative review of the choices of stem cell sources and hydrogels for cartilage tissue engineering. Ann. Transl. Med. 8 (23), 1598. 10.21037/atm-20-2342 33437797 PMC7791208

[B45] Deng Z. H. Li Y. S. Gao X. Lei G. Huard J. (2018). Bone morphogenetic proteins for articular cartilage regeneration. Osteoarthr. Cartil. 26 (9), 1153–1161. 10.1016/j.joca.2018.03.007 29580979

[B46] Dethe M. R. Prabakaran A. Ahmed H. Agrawal M. Roy U. Alexander A. (2022). PCL-PEG copolymer based injectable thermosensitive hydrogels. J. Control. Release 343, 217–236. 10.1016/j.jconrel.2022.01.035 35090961 PMC9134269

[B47] Dhand A. P. Galarraga J. H. Burdick J. A. (2021). Enhancing biopolymer hydrogel functionality through interpenetrating networks. Trends Biotechnol. 39 (5), 519–538. 10.1016/j.tibtech.2020.08.007 32950262 PMC7960570

[B48] Dimatteo R. Darling N. J. Segura T. (2018). *In situ* forming injectable hydrogels for drug delivery and wound repair. Adv. Drug Deliv. Rev. 127, 167–184. 10.1016/j.addr.2018.03.007 29567395 PMC6003852

[B49] Ding N. Li E. Ouyang X. Guo J. Wei B. (2021). The therapeutic potential of bone marrow mesenchymal stem cells for articular cartilage regeneration in osteoarthritis. Curr. Stem Cell Res. Ther. 16 (7), 840–847. 10.2174/1574888X16666210127130044 33504316

[B50] Echave M. C. Hernáez-Moya R. Iturriaga L. Pedraz J. L. Lakshminarayanan R. Dolatshahi-Pirouz A. (2019). Recent advances in gelatin-based therapeutics. Expert Opin. Biol. Ther. 19 (8), 773–779. 10.1080/14712598.2019.1610383 31009588

[B51] Elkhoury K. Morsink M. Sanchez-Gonzalez L. Kahn C. Tamayol A. Arab-Tehrany E. (2021). Biofabrication of natural hydrogels for cardiac, neural, and bone Tissue engineering Applications. Bioact. Mat. 6 (11), 3904–3923. 10.1016/j.bioactmat.2021.03.040 PMC8080408 33997485

[B52] Eslahi N. Abdorahim M. Simchi A. (2016). Smart polymeric hydrogels for cartilage tissue engineering: A review on the chemistry and biological functions. Biomacromolecules 17 (11), 3441–3463. 10.1021/acs.biomac.6b01235 27775329

[B53] Eyre D. R. (2004). Collagens and cartilage matrix homeostasis. Clin. Orthop. Relat. Res. 427 (Suppl. l), S118–S122. 10.1097/01.blo.0000144855.48640.b9 15480053

[B54] Fan W. Yuan L. Li J. Wang Z. Chen J. Guo C. (2020). Injectable double-crosslinked hydrogels with kartogenin-conjugated polyurethane nano-particles and transforming growth factor β3 for *in-situ* cartilage regeneration. Mater. Sci. Eng. C 110, 110705. 10.1016/j.msec.2020.110705 32204019

[B55] Farrell K. Joshi J. Kothapalli C. R. (2017). Injectable uncrosslinked biomimetic hydrogels as candidate scaffolds for neural stem cell delivery. J. Biomed. Mat. Res. A 105 (3), 790–805. 10.1002/jbm.a.35956 27798959

[B56] Fattahpour S. Shamanian M. Tavakoli N. Fathi M. Sadeghi-aliabadi H. Sheykhi S. R. (2020). An injectable carboxymethyl chitosan-methylcellulose-pluronic hydrogel for the encapsulation of meloxicam loaded nanoparticles. Int. J. Biol. Macromol. 151, 220–229. 10.1016/j.ijbiomac.2020.02.002 32027902

[B57] Flégeau K. Pace R. Gautier H. Rethore G. Guicheux J. Le Visage C. (2017). Toward the development of biomimetic injectable and macroporous biohydrogels for regenerative medicine. Adv. Colloid Interface Sci. 247, 589–609. 10.1016/j.cis.2017.07.012 28754381

[B58] Fu L. Li P. Zhu J. Liao Z. Gao C. Li H. (2022). Tetrahedral framework nucleic acids promote the biological functions and related mechanism of synovium-derived mesenchymal stem cells and show improved articular cartilage regeneration activity *in situ* . Bioact. Mat. 9, 411–427. 10.1016/j.bioactmat.2021.07.028 PMC8586787 34820580

[B59] Fu N. Dong T. Meng A. Meng Z. Zhu B. Lin Y. (2018). Research progress of the types and preparation techniques of scaffold materials in cartilage tissue engineering. Curr. Stem Cell Res. Ther. 13 (7), 583–590. 10.2174/1574888X12666170718152611 28721819

[B60] Fuggle N. R. Cooper C. Oreffo R. O. C. Price A. J. Kaux J. F. Maheu E. (2020). Alternative and complementary therapies in osteoarthritis and cartilage repair. Aging Clin. Exp. Res. 32 (4), 547–560. 10.1007/s40520-020-01515-1 32170710 PMC7170824

[B61] Gao Y. Li Z. Huang J. Zhao M. Wu J. (2020). *In situ* formation of injectable hydrogels for chronic wound healing. J. Mat. Chem. B 8 (38), 8768–8780. 10.1039/d0tb01074j 33026387

[B62] Gao Y. Liu S. Huang J. Guo W. Chen J. Zhang L. (2014). The ECM-cell interaction of cartilage extracellular matrix on chondrocytes. Biomed. Res. Int. 2014, 1–8. 10.1155/2014/648459 PMC4052144 24959581

[B63] García-Couce J. Tomás M. Fuentes G. Que I. Almirall A. Cruz L. J. (2022). Chitosan/Pluronic F127 thermosensitive hydrogel as an injectable dexamethasone delivery carrier. Gels 8 (1), 44. 10.3390/gels8010044 35049579 PMC8774693

[B64] García-Fernández L. Olmeda-Lozano M. Benito-Garzón L. Perez-Caballer A. San Roman J. Vazquez-Lasa B. (2020). Injectable hydrogel-based drug delivery system for cartilage regeneration. Mater. Sci. Eng. C 110, 110702. 10.1016/j.msec.2020.110702 32204016

[B65] Gomez-Florit M. Pardo A. Domingues R. M. A. Graca A. L. Babo P. S. Reis R. L. (2020). Natural-based hydrogels for tissue engineering applications. Molecules 25 (24), 5858. 10.3390/molecules25245858 PMC7763437 33322369

[B66] Gong D. Yu F. Zhou M. Dong W. Yan D. Zhang S. (2021). *Ex vivo* and *in vivo* properties of an injectable hydrogel derived from acellular ear cartilage extracellular matrix. Front. Bioeng. Biotechnol. 9, 740635. 10.3389/fbioe.2021.740635 34589475 PMC8474061

[B67] Gonzalez-Fernandez P. Rodríguez-Nogales C. Jordan O. Allemann E. (2022). Combination of mesenchymal stem cells and bioactive molecules in hydrogels for osteoarthritis treatment. Eur. J. Pharm. Biopharm. 172, 41–52. 10.1016/j.ejpb.2022.01.003 35114357

[B68] Gopinathan J. Noh I. (2018). Click chemistry-based injectable hydrogels and bioprinting inks for tissue engineering applications. Tissue Eng. Regen. Med. 15 (5), 531–546. 10.1007/s13770-018-0152-8 30603577 PMC6171698

[B69] Graça M. F. P. Miguel S. P. Cabral C. S. D. Correia I. J. (2020). Hyaluronic acid-based wound dressings: A review. Carbohydr. Polym. 241, 116364. 10.1016/j.carbpol.2020.116364 32507198

[B70] Grol M. W. Lee B. H. (2018). Gene therapy for repair and regeneration of bone and cartilage. Curr. Opin. Pharmacol. 40, 59–66. 10.1016/j.coph.2018.03.005 29621661

[B71] Guo Y. Gu J. Jiang Y. Zhou Y. Zhu Z. Ma T. (2021). Regulating the homogeneity of thiol-maleimide michael-type Addition-based hydrogels using amino biomolecules. Gels 7 (4), 206. 10.3390/gels7040206 34842701 PMC8628763

[B72] Gupta D. Gangwar A. Jyoti K. Sainaga Jyothi V. G. Sodhi R. K. Mehra N. K. (2020). Self healing hydrogels: A new paradigm immunoadjuvant for delivering peptide vaccine. Colloids Surfaces B Biointerfaces 194, 111171. 10.1016/j.colsurfb.2020.111171 32569887

[B73] Han Y. Yang J. Zhao W. Wang H. Sun Y. Chen Y. (2021). Biomimetic injectable hydrogel microspheres with enhanced lubrication and controllable drug release for the treatment of osteoarthritis. Bioact. Mat. 6 (10), 3596–3607. 10.1016/j.bioactmat.2021.03.022 PMC8022850 33869900

[B74] Hanafy A. S. El-Ganainy S. O. (2020). Thermoresponsive Hyalomer intra-articular hydrogels improve monoiodoacetate-induced osteoarthritis in rats. Int. J. Pharm. X. 573, 118859. 10.1016/j.ijpharm.2019.118859 31778752

[B75] Hassanpour M. Safardoust-Hojaghan H. Salavati-Niasari M. (2017). Degradation of methylene blue and Rhodamine B as water pollutants via green synthesized Co3O4/ZnO nanocomposite. J. Mol. Liq. 229, 293–299. 10.1016/j.molliq.2016.12.090

[B76] He C. Ji H. Qian Y. Wang Q. Liu X. Zhao W. (2019). Heparin-based and heparin-inspired hydrogels: Size-effect, gelation and biomedical applications. J. Mat. Chem. B 7 (8), 1186–1208. 10.1039/c8tb02671h 32255159

[B77] He P. Fu J. Wang D-A. (2016). Murine pluripotent stem cells derived scaffold-free cartilage grafts from a micro-cavitary hydrogel platform. Acta Biomater. 35, 87–97. 10.1016/j.actbio.2016.02.026 26911880

[B78] Heirani-Tabasi A. Hosseinzadeh S. Rabbani S. Ahmadi Tafti S. H. Jamshidi K. Soufizomorrod M. (2021). Cartilage tissue engineering by co-transplantation of chondrocyte extracellular vesicles and mesenchymal stem cells, entrapped in chitosan-hyaluronic acid hydrogel. Biomed. Mat. 16 (5), 055003. 10.1088/1748-605X/ac0cbf 34144542

[B79] Heo D. N. Kim H-J. Lee D. Lee S. J. Lee H. R. Kim H. (2020). Comparison of polysaccharides in articular cartilage regeneration associated with chondrogenic and autophagy-related gene expression. Int. J. Biol. Macromol. 146, 922–930. 10.1016/j.ijbiomac.2019.09.215 31726172

[B80] Hu C. Lu W. Mata A. Nishinari K. Fang Y. (2021). Ions-induced gelation of alginate: Mechanisms and applications. Int. J. Biol. Macromol. 177, 578–588. 10.1016/j.ijbiomac.2021.02.086 33617905

[B81] Hu M. Guo J. Du J. Liu Z. Li P. Ren X. (2019). Development of Ca2+-based, ion-responsive superabsorbent hydrogel for cement applications: Self-healing and compressive strength. J. Colloid Interface Sci. 538, 397–403. 10.1016/j.jcis.2018.12.004 30530077

[B82] Hu M. Yang J. Xu J. (2021). Structural and biological investigation of chitosan/hyaluronic acid with silanized-hydroxypropyl methylcellulose as an injectable reinforced interpenetrating network hydrogel for cartilage tissue engineering. Drug Deliv. (Lond). 28 (1), 607–619. 10.1080/10717544.2021.1895906 PMC7993376 33739203

[B83] Huang J. Liu F. Su H. Xiong J. Yang L. Xia J. (2022). Advanced nanocomposite hydrogels for cartilage tissue engineering. Gels 8 (2), 138. 10.3390/gels8020138 35200519 PMC8871651

[B84] Huang K. Li Q. Li Y. Yao Z. Luo D. Rao P. (2018). Cartilage tissue regeneration: The roles of cells, stimulating factors and scaffolds. Curr. Stem Cell Res. Ther. 13 (7), 547–567. 10.2174/1574888X12666170608080722 28595567

[B85] Huang Q. Zou Y. Arno M. C. Chen S. Wang T. Gao J. (2017). Hydrogel scaffolds for differentiation of adipose-derived stem cells. Chem. Soc. Rev. 46 (20), 6255–6275. 10.1039/c6cs00052e 28816316

[B86] Im G-I. Shin K-J. (2015). Epigenetic approaches to regeneration of bone and cartilage from stem cells. Expert Opin. Biol. Ther. 15 (2), 181–193. 10.1517/14712598.2015.960838 25283749

[B87] Ishihara M. Kishimoto S. Nakamura S. Sato Y. Hattori H. (2019). Polyelectrolyte complexes of natural polymers and their biomedical applications. Polym. (Basel) 11 (4), 672. 10.3390/polym11040672 PMC6523548 31013742

[B88] Jabbari E. Sepahvandi A. (2022). Decellularized articular cartilage microgels as microcarriers for expansion of mesenchymal stem cells. Gels 8 (3), 148. 10.3390/gels8030148 35323261 PMC8949257

[B89] Jaikumar D. Sajesh K. M. Soumya S. Nimal T. Chennazhi K. Nair S. V. (2015). Injectable alginate-O-carboxymethyl chitosan/nano fibrin composite hydrogels for adipose tissue engineering. Int. J. Biol. Macromol. 74, 318–326. 10.1016/j.ijbiomac.2014.12.037 25544040

[B90] Jalani G. Rosenzweig D. H. Makhoul G. Abdalla S. Cecere R. Vetrone F. (2015). Tough, *in-situ* thermogelling, injectable hydrogels for biomedical applications. Macromol. Biosci. 15 (4), 473–480. 10.1002/mabi.201400406 25557500

[B91] Jeznach O. Kołbuk D. Sajkiewicz P. (2018). Injectable hydrogels and nanocomposite hydrogels for cartilage regeneration. J. Biomed. Mat. Res. A 106 (10), 2762–2776. 10.1002/jbm.a.36449 29726104

[B92] Ji X. Lei Z. Yuan M. Zhu H. Yuan X. Liu W. (2020). Cartilage repair mediated by thermosensitive photocrosslinkable TGFβ1-loaded GM-HPCH via immunomodulating macrophages, recruiting MSCs and promoting chondrogenesis. Theranostics 10 (6), 2872–2887. 10.7150/thno.41622 32194841 PMC7052899

[B93] Jian F. Zhang Y. Wang J. Ba K. Mao R. Lai W. (2012). Toxicity of biodegradable nanoscale preparations. Curr. Drug Metab. 13 (4), 440–446. 10.2174/138920012800166517 22443538

[B94] Jiang T. Yang T. Bao Q. Sun W. Yang M. Mao C. (2021). Construction of tissue-customized hydrogels from cross-linkable materials for effective tissue regeneration. J. Mat. Chem. B 2021. 10.1039/d1tb01935j 34812829

[B95] Jiang Y. Cai Y. Zhang W. Yin Z. Hu C. Tong T. (2016). Human cartilage-derived progenitor cells from committed chondrocytes for efficient cartilage repair and regeneration. Stem Cells Transl. Med. 5 (6), 733–744. 10.5966/sctm.2015-0192 27130221 PMC4878331

[B96] Jiang Y. Tuan R. S. (2015). Origin and function of cartilage stem/progenitor cells in osteoarthritis. Nat. Rev. Rheumatol. 11 (4), 206–212. 10.1038/nrrheum.2014.200 25536487 PMC5413931

[B97] Jin R. Teixeira L. S. M. Dijkstra P. J. van Blitterswijk C. Karperien M. Feijen J. (2010). Enzymatically-crosslinked injectable hydrogels based on biomimetic dextran-hyaluronic acid conjugates for cartilage tissue engineering. Biomaterials 31 (11), 3103–3113. 10.1016/j.biomaterials.2010.01.013 20116847

[B98] Jin Y. Koh R. H. Kim S-H. Kim K. M. Park G. K. Hwang N. S. (2020). Injectable anti-inflammatory hyaluronic acid hydrogel for osteoarthritic cartilage repair. Mater. Sci. Eng. C 115, 111096. 10.1016/j.msec.2020.111096 32600700

[B99] Johnstone B. Stoddart M. J. Im G-I. (2020). Multi-disciplinary approaches for cell-based cartilage regeneration. J. Orthop. Res. 38 (3), 463–472. 10.1002/jor.24458 31478253

[B100] Jonidi Shariatzadeh F. Solouk A. Bagheri Khoulenjani S. Bonakdar S. Mirzadeh H. (2021). Injectable and reversible preformed cryogels based on chemically crosslinked gelatin methacrylate (GelMA) and physically crosslinked hyaluronic acid (HA) for soft tissue engineering. Colloids Surfaces B Biointerfaces 203, 111725. 10.1016/j.colsurfb.2021.111725 33838583

[B101] Kahn J. S. Hu Y. Willner I. (2017). Stimuli-responsive DNA-based hydrogels: From basic principles to applications. Acc. Chem. Res. 50 (4), 680–690. 10.1021/acs.accounts.6b00542 28248486

[B102] Katz J. N. Arant K. R. Loeser R. F. (2021). Diagnosis and treatment of hip and knee osteoarthritis: A review. JAMA 325 (6), 568. 10.1001/jama.2020.22171 33560326 PMC8225295

[B103] Khajouei S. Ravan H. Ebrahimi A. (2020). DNA hydrogel-empowered biosensing. Adv. Colloid Interface Sci. 275, 102060. 10.1016/j.cis.2019.102060 31739981 PMC7094116

[B104] Khan F. Atif M. Haseen M. Kamal S. Khan M. S. Shahid S. (2022). Synthesis, classification and properties of hydrogels: Their applications in drug delivery and agriculture. J. Mat. Chem. B 10 (2), 170–203. 10.1039/d1tb01345a 34889937

[B105] Kim D. Y. Park H. Kim S. W. Lee J. W. Lee K. Y. (2017). Injectable hydrogels prepared from partially oxidized hyaluronate and glycol chitosan for chondrocyte encapsulation. Carbohydr. Polym. 157, 1281–1287. 10.1016/j.carbpol.2016.11.002 27987834

[B106] Kim I. L. Mauck R. L. Burdick J. A. (2011). Hydrogel design for cartilage tissue engineering: A case study with hyaluronic acid. Biomaterials 32 (34), 8771–8782. 10.1016/j.biomaterials.2011.08.073 21903262 PMC3183132

[B107] Kim Y. S. Mehta S. M. Guo J. L. Pearce H. A. Smith B. T. Watson E. (2021). Evaluation of tissue integration of injectable, cell-laden hydrogels of cocultures of mesenchymal stem cells and articular chondrocytes with an *ex vivo* cartilage explant model. Biotechnol. Bioeng. 118 (8), 2958–2966. 10.1002/bit.27804 33913514

[B108] Kouser R. Vashist A. Zafaryab M. Rizvi M. A. Ahmad S. (2018). Biocompatible and mechanically robust nanocomposite hydrogels for potential applications in tissue engineering. Mater. Sci. Eng. C 84, 168–179. 10.1016/j.msec.2017.11.018 29519426

[B109] Krishnan Y. Grodzinsky A. J. (2018). Cartilage diseases. Matrix Biol. 71-72, 51–69. 10.1016/j.matbio.2018.05.005 29803938 PMC6146013

[B110] Kwon H. Brown W. E. Lee C. A. Wang D. Paschos N. Hu J. C. (2019). Surgical and tissue engineering strategies for articular cartilage and meniscus repair. Nat. Rev. Rheumatol. 15 (9), 550–570. 10.1038/s41584-019-0255-1 31296933 PMC7192556

[B111] Lee C. O'Connell C. D. Onofrillo C. Choong P. F. M. Di Bella C. Duchi S. (2020). Human articular cartilage repair: Sources and detection of cytotoxicity and genotoxicity in photo-crosslinkable hydrogel bioscaffolds. Stem Cells Transl. Med. 9 (3), 302–315. 10.1002/sctm.19-0192 31769213 PMC7031631

[B112] Lee K. Y. Mooney D. J. (2012). Alginate: Properties and biomedical applications. Prog. Polym. Sci. 37 (1), 106–126. 10.1016/j.progpolymsci.2011.06.003 22125349 PMC3223967

[B113] Lee S. Choi J. Youn J. Lee Y. Kim W. Choe S. (2021). Development and evaluation of gellan gum/silk fibroin/chondroitin sulfate ternary injectable hydrogel for cartilage tissue engineering. Biomolecules 11 (8), 1184. 10.3390/biom11081184 34439850 PMC8394129

[B114] Lei Y. Wang Y. Shen J. Cai Z. Zhao C. Chen H. (2022). Injectable hydrogel microspheres with self-renewable hydration layers alleviate osteoarthritis. Sci. Adv. 8 (5), eabl6449. 10.1126/sciadv.abl6449 35108047 PMC8809544

[B115] Li F. Lyu D. Liu S. Guo W. (2020). DNA hydrogels and microgels for biosensing and biomedical applications. Adv. Mat. 32 (3), e1806538. 10.1002/adma.201806538 31379017

[B116] Li J. Chen G. Xu X. Abdou P. Jiang Q. Shi D. (2019). Advances of injectable hydrogel-based scaffolds for cartilage regeneration. Regen. Biomater. 6 (3), 129–140. 10.1093/rb/rbz022 31198581 PMC6547311

[B117] Li J. Lai Y. Li M. Chen X. Zhou M. Wang W. (2022). Repair of infected bone defect with Clindamycin-Tetrahedral DNA nanostructure Complex-loaded 3D bioprinted hybrid scaffold. Chem. Eng. J. 435, 134855. 10.1016/j.cej.2022.134855

[B118] Li J. Liu N. Huang Z. Wang W. Hou D. Wang W. (2021). Intra-articular injection of loaded sPL sustained-release microspheres inhibits osteoarthritis and promotes cartilaginous repairs. J. Orthop. Surg. Res. 16 (1), 646. 10.1186/s13018-021-02777-9 34717689 PMC8557014

[B119] Li X. Sigen A. Xu Q. Alshehri F. Zeng M. Zhou D. (2020). Cartilage-derived progenitor cell-laden injectable hydrogel-an approach for cartilage tissue regeneration. ACS Appl. Bio Mat. 3 (8), 4756–4765. 10.1021/acsabm.0c00294 35021723

[B120] Li X. Lu Y. Wang Y. Zhou S. Li L. Zhao F. (2021). Thermo-responsive injectable naringin-loaded hydrogel polymerised sodium alginate/bioglass delivery for articular cartilage. Drug Deliv. (Lond). 28 (1), 1290–1300. 10.1080/10717544.2021.1938752 PMC8238061 34176372

[B121] Li X. Xu Q. Johnson M. Wang X. Lyu J. Li Y. (2021). A chondroitin sulfate based injectable hydrogel for delivery of stem cells in cartilage regeneration. Biomater. Sci. 9 (11), 4139–4148. 10.1039/d1bm00482d 33955435

[B122] Li Y. Rodrigues J. Tomás H. (2012). Injectable and biodegradable hydrogels: Gelation, biodegradation and biomedical applications. Chem. Soc. Rev. 41 (6), 2193–2221. 10.1039/c1cs15203c 22116474

[B123] Li Y. Wang X. Han Y. Sun H. Y. Hilborn J. Shi L. (2021). Click chemistry-based biopolymeric hydrogels for regenerative medicine. Biomed. Mat. 16 (2), 022003. 10.1088/1748-605X/abc0b3 33049725

[B124] Lim M. H. Jeun J. H. Kim D. H. Park S. H. Kim S. J. Lee W. S. (2020). Evaluation of collagen gel-associated human nasal septum-derived chondrocytes as a clinically applicable injectable therapeutic agent for cartilage repair. Tissue Eng. Regen. Med. 17 (3), 387–399. 10.1007/s13770-020-00261-9 32399775 PMC7260351

[B125] Lin S-J. Chan Y-C. Su Z-C. Yeh W. Lai P. Chu I. (2021). Growth factor-loaded microspheres in mPEG-polypeptide hydrogel system for articular cartilage repair. J. Biomed. Mat. Res. A 109 (12), 2516–2526. 10.1002/jbm.a.37246 34190399

[B126] Lin X. Tsao C. T. Kyomoto M. Zhang M. (2021). Injectable natural polymer hydrogels for treatment of knee osteoarthritis. Adv. Healthc. Mat. 11, e2101479. 10.1002/adhm.202101479 34535978

[B127] Liu H-Y. Lin C-C. (2019). A diffusion-reaction model for predicting enzyme-mediated dynamic hydrogel stiffening. Gels 5 (1), 17. 10.3390/gels5010017 PMC6473751 30871250

[B128] Liu M. Zeng X. Ma C. Yi H. Ali Z. Mou X. (2017). Injectable hydrogels for cartilage and bone tissue engineering. Bone Res. 5, 17014. 10.1038/boneres.2017.14 28584674 PMC5448314

[B129] Liu R. Zhang S. Chen X. (2020). Injectable hydrogels for tendon and ligament tissue engineering. J. Tissue Eng. Regen. Med. 14 (9), 1333–1348. 10.1002/term.3078 32495524

[B130] Liu X. Liu S. Yang R. Wang P. Zhang W. Tan X. (2021). Gradient chondroitin sulfate/poly (γ-glutamic acid) hydrogels inducing differentiation of stem cells for cartilage tissue engineering. Carbohydr. Polym. 270, 118330. 10.1016/j.carbpol.2021.118330 34364592

[B131] Liu X. Yang Y. Li Y. Niu X. Zhao B. Wang Y. (2017). Integration of stem cell-derived exosomes with *in situ* hydrogel glue as a promising tissue patch for articular cartilage regeneration. Nanoscale 9 (13), 4430–4438. 10.1039/c7nr00352h 28300264

[B132] Liu Y. Hsu S-H. (2018). Synthesis and biomedical applications of self-healing hydrogels. Front. Chem. 6, 449. 10.3389/fchem.2018.00449 30333970 PMC6176467

[B133] Liu Y. Wang M. Luo Y. Liang Q. Yu Y. Chen F. (2021). Enhancing stem cell therapy for cartilage repair in osteoarthritis-A hydrogel focused approach. Gels 7 (4), 263. 10.3390/gels7040263 34940323 PMC8701810

[B134] Lokhande G. Carrow J. K. Thakur T. Xavier J. R. Parani M. Bayless K. J. (2018). Nanoengineered injectable hydrogels for wound healing application. Acta Biomater. 70, 35–47. 10.1016/j.actbio.2018.01.045 29425720 PMC7499308

[B135] Lu L. Yuan S. Wang J. Shen Y. Deng S. Xie L. (2018). the formation mechanism of hydrogels. Curr. Stem Cell Res. Ther. 13 (7), 490–496. 10.2174/1574888X12666170612102706 28606044

[B136] Luo J. Zhang Y. Zhu S. Tong Y. Ji L. Zhang W. (2021). The application prospect of metal/metal oxide nanoparticles in the treatment of osteoarthritis. Schmiedeb. Arch. Pharmacol. 394 (10), 1991–2002. 10.1007/s00210-021-02131-0 PMC8486704 34415355

[B137] Luu C. H. Nguyen G. Le T-T. Nguyen T. M. N. Giang Phan V. H. Murugesan M. (2022). Graphene oxide-reinforced alginate hydrogel for controlled release of local anesthetics: Synthesis, characterization, and release studies. Gels 8 (4), 246. 10.3390/gels8040246 35448147 PMC9026710

[B138] Lynch B. Crawford K. Baruti O. Abdulahad A. Webster M. Puetzer J. (2017). The effect of hypoxia on thermosensitive poly(N-vinylcaprolactam) hydrogels with tunable mechanical integrity for cartilage tissue engineering. J. Biomed. Mat. Res. 105 (7), 1863–1873. 10.1002/jbm.b.33705 27240310

[B139] Ma Q. Liao J. Cai X. (2018). Different sources of stem cells and their application in cartilage tissue engineering. Curr. Stem Cell Res. Ther. 13 (7), 568–575. 10.2174/1574888X13666180122151909 29359678

[B140] Madry H. Cucchiarini M. (2016). Gene therapy for human osteoarthritis: Principles and clinical translation. Expert Opin. Biol. Ther. 16 (3), 331–346. 10.1517/14712598.2016.1124084 26593049

[B141] Mahajan A. Singh A. Datta D. Katti D. S. (2022). Bioinspired injectable hydrogels dynamically stiffen and contract to promote mechanosensing-mediated chondrogenic commitment of stem cells. ACS Appl. Mat. Interfaces 14 (6), 7531–7550. 10.1021/acsami.1c11840 35119254

[B142] Mang T. Kleinschmidt-Dörr K. Ploeger F. Lindemann S. Gigout A. (2020). The GDF-5 mutant M1673 exerts robust anabolic and anti-catabolic effects in chondrocytes. J. Cell. Mol. Med. 24 (13), 7141–7150. 10.1111/jcmm.15149 32497388 PMC7339174

[B143] Mantooth S. M. Munoz-Robles B. G. Webber M. J. (2019). Dynamic hydrogels from host-guest supramolecular interactions. Macromol. Biosci. 19 (1), e1800281. 10.1002/mabi.201800281 30303631

[B144] Martin A. R. Patel J. M. Locke R. C. Eby M. R. Saleh K. S. Davidson M. D. (2021). Nanofibrous hyaluronic acid scaffolds delivering TGF-β3 and SDF-1α for articular cartilage repair in a large animal model. Acta Biomater. 126, 170–182. 10.1016/j.actbio.2021.03.013 33753316 PMC8314069

[B145] Mathew A. P. Uthaman S. Cho K-H. Cho C. S. Park I. K. (2018). Injectable hydrogels for delivering biotherapeutic molecules. Int. J. Biol. Macromol. 110, 17–29. 10.1016/j.ijbiomac.2017.11.113 29169942

[B146] Matricardi P. Di Meo C. Coviello T. Hennink W. E. Alhaique F. (2013). Interpenetrating Polymer Networks polysaccharide hydrogels for drug delivery and tissue engineering. Adv. Drug Deliv. Rev. 65 (9), 1172–1187. 10.1016/j.addr.2013.04.002 23603210

[B147] Mazini L. Rochette L. Amine M. Malka G. (2019). Regenerative capacity of adipose derived stem cells (ADSCs), comparison with mesenchymal stem cells (MSCs). Int. J. Mol. Sci. 20 (10), 2523. 10.3390/ijms20102523 PMC6566837 31121953

[B148] Mehrali M. Thakur A. Pennisi C. P. Talebian S. Arpanaei A. Nikkhah M. (2017). Nanoreinforced hydrogels for tissue engineering: Biomaterials that are compatible with load-bearing and electroactive tissues. Adv. Mat. 29 (8), 1603612. 10.1002/adma.201603612 27966826

[B149] Meng W. Gao L. Venkatesan J. K. Wang G. Madry H. Cucchiarini M. (2019). Translational applications of photopolymerizable hydrogels for cartilage repair. J. Exp. Orthop. 6 (1), 47. 10.1186/s40634-019-0215-3 31807962 PMC6895316

[B150] Mohamed-Ahmed S. Fristad I. Lie S. A. Suliman S. Mustafa K. Vindenes H. (2018). Adipose-derived and bone marrow mesenchymal stem cells: A donor-matched comparison. Stem Cell Res. Ther. 9 (1), 168. 10.1186/s13287-018-0914-1 29921311 PMC6008936

[B151] Mok S-W. Fu S-C. Cheuk Y-C. Chu I. M. Chan K. M. Qin L. (2020). Intra-articular delivery of quercetin using thermosensitive hydrogel attenuate cartilage degradation in an osteoarthritis rat model. Cartilage 11 (4), 490–499. 10.1177/1947603518796550 30160166 PMC7488941

[B152] Monsef R. Salavati-Niasari M. (2021). Hydrothermal architecture of Cu_5_V_2_O_10_ nanostructures as new electro-sensing catalysts for voltammetric quantification of mefenamic acid in pharmaceuticals and biological samples. Biosens. Bioelectron. 178, 113017. 10.1016/j.bios.2021.113017 33493895

[B153] Motahari F. Mozdianfard M. R. Salavati-Niasari M. (2015). Synthesis and adsorption studies of NiO nanoparticles in the presence of H2acacen ligand, for removing Rhodamine B in wastewater treatment. Process Saf. Environ. Prot. 93, 282–292. 10.1016/j.psep.2014.06.006

[B154] Motealleh A. Kehr N. S. (2017). Nanocomposite hydrogels and their applications in tissue engineering. Adv. Healthc. Mat. 6 (1), 1600938. 10.1002/adhm.201600938 27900856

[B155] Muir V. G. Burdick J. A. (2021). Chemically modified biopolymers for the formation of biomedical hydrogels. Chem. Rev. 121 (18), 10908–10949. 10.1021/acs.chemrev.0c00923 33356174 PMC8943712

[B156] Muscolino E. Di Stefano A. B. Trapani M. Sabatino M. A. Giacomazza D. Moschella F. (2021). Injectable xyloglucan hydrogels incorporating spheroids of adipose stem cells for bone and cartilage regeneration. Mater. Sci. Eng. C 131, 112545. 10.1016/j.msec.2021.112545 34857257

[B157] Naahidi S. Jafari M. Logan M. Wang Y. Yuan Y. Bae H. (2017). Biocompatibility of hydrogel-based scaffolds for tissue engineering applications. Biotechnol. Adv. 35 (5), 530–544. 10.1016/j.biotechadv.2017.05.006 28558979

[B158] Nagahama K. Oyama N. Ono K. Hotta A. Kawauchi K. Nishikata T. (2018). Nanocomposite injectable gels capable of self-replenishing regenerative extracellular microenvironments for *in vivo* tissue engineering. Biomater. Sci. 6 (3), 550–561. 10.1039/c7bm01167a 29379910

[B159] Ngadimin K. D. Stokes A. Gentile P. Ferreira A. M. (2021). Biomimetic hydrogels designed for cartilage tissue engineering. Biomater. Sci. 9 (12), 4246–4259. 10.1039/d0bm01852j 33710205

[B160] Nguyen T. P. T. Li F. Shrestha S. Tuan R. S. Thissen H. Forsythe J. S. (2021). Cell-laden injectable microgels: Current status and future prospects for cartilage regeneration. Biomaterials 279, 121214. 10.1016/j.biomaterials.2021.121214 34736147

[B161] Nicol E. (2021). Photopolymerized porous hydrogels. Biomacromolecules 22 (4), 1325–1345. 10.1021/acs.biomac.0c01671 33793224

[B162] Niemczyk B. Sajkiewicz P. Kolbuk D. (2018). Injectable hydrogels as novel materials for central nervous system regeneration. J. Neural Eng. 15 (5), 051002. 10.1088/1741-2552/aacbab 29889043

[B163] Oliva N. Conde J. Wang K. Artzi N. (2017). Designing hydrogels for on-demand therapy. Acc. Chem. Res. 50 (4), 669–679. 10.1021/acs.accounts.6b00536 28301139 PMC6527116

[B164] Oliveira I. M. Gonçalves C. Shin M. E. Lee S. Reis R. L. Khang G. (2021). Enzymatically crosslinked tyramine-gellan gum hydrogels as drug delivery system for rheumatoid arthritis treatment. Drug Deliv. Transl. Res. 11 (3), 1288–1300. 10.1007/s13346-020-00855-9 32924098

[B165] Oliveira J. T. Gardel L. S. Rada T. Martins L. Gomes M. E. Reis R. L. (2010). Injectable gellan gum hydrogels with autologous cells for the treatment of rabbit articular cartilage defects. J. Orthop. Res. 28 (9), 1193–1199. 10.1002/jor.21114 20187118

[B166] Onofrillo C. Duchi S. Francis S. O'Connell C. D. Caballero Aguilar L. M. Doyle S. (2021). Flash: Fluorescently LAbelled Sensitive Hydrogel to monitor bioscaffolds degradation during neocartilage generation. Biomaterials 264, 120383. 10.1016/j.biomaterials.2020.120383 33099133

[B167] Panyamao P. Ruksiriwanich W. Sirisa-Ard P. Charumanee S. (2020). Injectable thermosensitive chitosan/pullulan-based hydrogels with improved mechanical properties and swelling capacity. Polym. (Basel) 12 (11), 2514. 10.3390/polym12112514 PMC7692642 33126695

[B168] Park K. M. Lee S. Y. Joung Y. K. Na J. S. Park K. D. (2009). Thermosensitive chitosan-Pluronic hydrogel as an injectable cell delivery carrier for cartilage regeneration. Acta Biomater. 5 (6), 1956–1965. 10.1016/j.actbio.2009.01.040 19261553

[B169] Pascual-Garrido C. Rodriguez-Fontan F. Aisenbrey E. A. Payne K. A. Chahla J. Goodrich L. R. (2018). Current and novel injectable hydrogels to treat focal chondral lesions: Properties and applicability. J. Orthop. Res. 36 (1), 64–75. 10.1002/jor.23760 28975658 PMC5839960

[B170] Peng L. Zhou Y. Lu W. Zhu W. Li Y. Chen K. (2019). Characterization of a novel polyvinyl alcohol/chitosan porous hydrogel combined with bone marrow mesenchymal stem cells and its application in articular cartilage repair. BMC Musculoskelet. Disord. 20 (1), 257. 10.1186/s12891-019-2644-7 31138200 PMC6540438

[B171] Pereira R. C. Scaranari M. Castagnola P. Grandizio M. Azevedo H. S. Reis R. L. (2009). Novel injectable gel (system) as a vehicle for human articular chondrocytes in cartilage tissue regeneration. J. Tissue Eng. Regen. Med. 3 (2), 97–106. 10.1002/term.145 19172577

[B172] Piantanida E. Alonci G. Bertucci A. De Cola L. (2019). Design of nanocomposite injectable hydrogels for minimally invasive surgery. Acc. Chem. Res. 52 (8), 2101–2112. 10.1021/acs.accounts.9b00114 31291090

[B173] Pierau L. Versace D-L. (2021). Light and hydrogels: A new generation of antimicrobial materials. Mater. (Basel) 14 (4), 787. 10.3390/ma14040787 PMC7915775 33562335

[B174] Piras C. C. Smith D. K. (2020). Multicomponent polysaccharide alginate-based bioinks. J. Mat. Chem. B 8 (36), 8171–8188. 10.1039/d0tb01005g 32776063

[B175] Poddar S. K. Widstrom L. (2017). Nonoperative options for management of articular cartilage disease. Clin. Sports Med. 36 (3), 447–456. 10.1016/j.csm.2017.02.003 28577705

[B176] Pogue R. Lyons K. (2006). BMP signaling in the cartilage growth plate. Curr. Top. Dev. Biol. 76, 1–48. 10.1016/S0070-2153(06)76001-X 17118262

[B177] Pupkaite J. Rosenquist J. Hilborn J. Samanta A. (2019). Injectable shape-holding collagen hydrogel for cell encapsulation and delivery cross-linked using thiol-michael addition click reaction. Biomacromolecules 20 (9), 3475–3484. 10.1021/acs.biomac.9b00769 31408340

[B178] Qi C. Liu J. Jin Y. Xu L. Wang G. Wang Z. (20182021). Corrigendum to “Photo-crosslinkable, injectable sericin hydrogel as 3D biomimetic extracellular matrix for minimally invasive repairing cartilage” [biomaterials 163 (2018) 89–104]. Biomaterials 278, 121134. 10.1016/j.biomaterials.2021.121134 29455069

[B179] Qi C. Liu J. Jin Y. Xu L. Wang G. Wang Z. (2018). Photo-crosslinkable, injectable sericin hydrogel as 3D biomimetic extracellular matrix for minimally invasive repairing cartilage. Biomaterials 163, 89–104. 10.1016/j.biomaterials.2018.02.016 29455069

[B180] Quadrado R. F. N. Macagnan K. L. Moreira A. S. Fajardo A. R. (2021). Chitosan-based hydrogel crosslinked through an aza-Michael addition catalyzed by boric acid. Int. J. Biol. Macromol. 193 (Pt B), 1032–1042. 10.1016/j.ijbiomac.2021.11.075 34800516

[B181] Radhakrishnan J. Krishnan U. M. Sethuraman S. (2014). Hydrogel based injectable scaffolds for cardiac tissue regeneration. Biotechnol. Adv. 32 (2), 449–461. 10.1016/j.biotechadv.2013.12.010 24406815

[B182] Ravari M. K. Mashayekhan S. Zarei F. Sayyahpour F. A. Taghiyar L. Baghban Eslaminejad M. (2021). Fabrication and characterization of an injectable reinforced composite scaffold for cartilage tissue engineering: An *in vitro* study. Biomed. Mat. 16 (4), 045007. 10.1088/1748-605X/abed97 33784250

[B183] Ren K. He C. Xiao C. Li G. Chen X. (2015). Injectable glycopolypeptide hydrogels as biomimetic scaffolds for cartilage tissue engineering. Biomaterials 51, 238–249. 10.1016/j.biomaterials.2015.02.026 25771014

[B184] Rigogliuso S. Salamone M. Barbarino E. Nicosia A. Ghersi G. (2020). Production of injectable marine collagen-based hydrogel for the maintenance of differentiated chondrocytes in tissue engineering applications. Int. J. Mol. Sci. 21 (16), 5798. 10.3390/ijms21165798 PMC7461064 32806778

[B185] Riley L. Schirmer L. Segura T. (2019). Granular hydrogels: emergent properties of jammed hydrogel microparticles and their applications in tissue repair and regeneration. Curr. Opin. Biotechnol. 60, 1–8. 10.1016/j.copbio.2018.11.001 30481603 PMC6534490

[B186] Rinoldi C. Lanzi M. Fiorelli R. Nakielski P. Zembrzycki K. Kowalewski T. (2021). Three-dimensional printable conductive semi-interpenetrating polymer network hydrogel for neural tissue applications. Biomacromolecules 22 (7), 3084–3098. 10.1021/acs.biomac.1c00524 34151565 PMC8462755

[B187] Sahajpal K. Shekhar S. Kumar A. Sharma B. Meena M. K. Bhagi A. K. (2022). Dynamic protein and polypeptide hydrogels based on Schiff base co-assembly for biomedicine. J. Mat. Chem. B 10 (17), 3173–3198. 10.1039/d2tb00077f 35352081

[B188] Sala R. L. Kwon M. Y. Kim M. Gullbrand S. E. Henning E. A. Mauck R. L. (2017). Thermosensitive poly(N-vinylcaprolactam) injectable hydrogels for cartilage tissue engineering. Tissue Eng. Part A 23 (17-18), 935–945. 10.1089/ten.tea.2016.0464 28384053 PMC5610396

[B189] Salavati-Niasari M. Davar F. (2006). *In situ* one-pot template synthesis (IOPTS) and characterization of copper(II) complexes of 14-membered hexaaza macrocyclic ligand “3, 10-dialkyl-dibenzo-1, 3, 5, 8, 10, 12-hexaazacyclotetradecane”. Inorg. Chem. Commun. 9 (2), 175–179. 10.1016/j.inoche.2005.10.028

[B190] Saravanan S. Vimalraj S. Thanikaivelan P. Banudevi S. Manivasagam G. (2019). A review on injectable chitosan/beta glycerophosphate hydrogels for bone tissue regeneration. Int. J. Biol. Macromol. 121, 38–54. 10.1016/j.ijbiomac.2018.10.014 30291931

[B191] Saveh-Shemshaki N. Nair S. L. Laurencin C. T. (2019). Nanofiber-based matrices for rotator cuff regenerative engineering. Acta Biomater. 94, 64–81. 10.1016/j.actbio.2019.05.041 31128319

[B192] Schaeffer C. Pfaff B. N. Cornell N. J. Salopek L. S. Shan S. Viyar J. (2020). Injectable microannealed porous scaffold for articular cartilage regeneration. Ann. Plast. Surg. 84 (6S Suppl. 5), S446–S450. 10.1097/SAP.0000000000002271 32032122

[B193] Seo B-B. Kwon Y. Kim J. Hong K. H. Kim S. E. Song H. R. (2022). Injectable polymeric nanoparticle hydrogel system for long-term anti-inflammatory effect to treat osteoarthritis. Bioact. Mat. 7, 14–25. 10.1016/j.bioactmat.2021.05.028 PMC8377411 34466714

[B194] Sevastianov V. I. Basok Y. B. Kirsanova L. A. Grigoriev A. M. Kirillova A. D. Nemets E. A. (2021). A comparison of the capacity of mesenchymal stromal cells for cartilage regeneration depending on collagen-based injectable biomimetic scaffold type. Life (Basel) 11 (8), 756. 10.3390/life11080756 34440500 PMC8400656

[B195] Shao J. Zhang W. Yang T. (2015). Using mesenchymal stem cells as a therapy for bone regeneration and repairing. Biol. Res. 48, 62. 10.1186/s40659-015-0053-4 26530042 PMC4630918

[B196] Shi J. Yu L. Ding J. (2021). PEG-based thermosensitive and biodegradable hydrogels. Acta Biomater. 128, 42–59. 10.1016/j.actbio.2021.04.009 33857694

[B197] Shi W. Fang F. Kong Y. Greer S. E. Kuss M. Liu B. (2021). Dynamic hyaluronic acid hydrogel with covalent linked gelatin as an anti-oxidative bioink for cartilage tissue engineering. Biofabrication 14 (1), 014107. 10.1088/1758-5090/ac42de 34905737

[B198] Shojarazavi N. Mashayekhan S. Pazooki H. Mohsenifard S. Baniasadi H. (2021). Alginate/cartilage extracellular matrix-based injectable interpenetrating polymer network hydrogel for cartilage tissue engineering. J. Biomater. Appl. 36 (5), 803–817. 10.1177/08853282211024020 34121491

[B199] Simon T. M. Jackson D. W. (2006). Articular cartilage: Injury pathways and treatment options. Sports Med. Arthrosc. Rev. 14 (3), 146–154. 10.1097/00132585-200609000-00006 17135961

[B200] Sirong S. Yang C. Taoran T. Songhang L. Shiyu L. Yuxin Z. (2020). Effects of tetrahedral framework nucleic acid/wogonin complexes on osteoarthritis. Bone Res. 8, 6. 10.1038/s41413-019-0077-4 32047705 PMC7010777

[B201] Skopinska-Wisniewska J. De la Flor S. Kozlowska J. (2021). From supramolecular hydrogels to multifunctional carriers for biologically active substances. Int. J. Mol. Sci. 22 (14), 7402. 10.3390/ijms22147402 34299020 PMC8307912

[B202] Song F. Li X. Wang Q. Liao L. Zhang C. (2015). Nanocomposite hydrogels and their applications in drug delivery and tissue engineering. J. Biomed. Nanotechnol. 11 (1), 40–52. 10.1166/jbn.2015.1962 26301299

[B203] Song H. Zhao J. Cheng J. Feng Z. Wang J. Momtazi‐Borojeni A. A. (2021). Extracellular vesicles in chondrogenesis and cartilage regeneration. J. Cell. Mol. Med. 25 (11), 4883–4892. 10.1111/jcmm.16290 33942981 PMC8178250

[B204] Stampoultzis T. Karami P. Pioletti D. P. (2021). Thoughts on cartilage tissue engineering: A 21st century perspective. Curr. Res. Transl. Med. 69 (3), 103299. 10.1016/j.retram.2021.103299 34192658

[B205] Sun A. X. Lin H. Fritch M. R. Shen H. Alexander P. G. DeHart M. (2017). Chondrogenesis of human bone marrow mesenchymal stem cells in 3-dimensional, photocrosslinked hydrogel constructs: Effect of cell seeding density and material stiffness. Acta Biomater. 58, 302–311. 10.1016/j.actbio.2017.06.016 28611002 PMC5813286

[B206] Sun K. Guo J. Yao X. Guo Z. Guo F. (2021). Growth differentiation factor 5 in cartilage and osteoarthritis: A possible therapeutic candidate. Cell Prolif. 54 (3), e12998. 10.1111/cpr.12998 33522652 PMC7941218

[B207] Suo H. Li L. Zhang C. Yin J. Xu K. Liu J. (2020). Glucosamine-grafted methacrylated gelatin hydrogels as potential biomaterials for cartilage repair. J. Biomed. Mat. Res. 108 (3), 990–999. 10.1002/jbm.b.34451 31369700

[B208] Talaat W. Aryal Ac S. Al Kawas S. Samsudin A. R. Kandile N. G. Harding D. R. (2020). Nanoscale thermosensitive hydrogel scaffolds promote the chondrogenic differentiation of dental pulp stem and progenitor cells: A minimally invasive approach for cartilage regeneration. Int. J. Nanomedicine 15, 7775–7789. 10.2147/IJN.S274418 33116500 PMC7567564

[B209] Tang Q. Lim T. Shen L-Y. Zheng G. Wei X. J. Zhang C. Q. (2021). Well-dispersed platelet lysate entrapped nanoparticles incorporate with injectable PDLLA-PEG-PDLLA triblock for preferable cartilage engineering application. Biomaterials 268, 120605. 10.1016/j.biomaterials.2020.120605 33360073

[B210] Tao S-C. Huang J-Y. Gao Y. Li Z. X. Wei Z. Y. Dawes H. (2021). Small extracellular vesicles in combination with sleep-related circRNA3503: A targeted therapeutic agent with injectable thermosensitive hydrogel to prevent osteoarthritis. Bioact. Mat. 6 (12), 4455–4469. 10.1016/j.bioactmat.2021.04.031 PMC8120802 34027234

[B211] Teixeira L. S. M. Feijen J. van Blitterswijk C. A. Dijkstra P. J. Karperien M. (2012). Enzyme-catalyzed crosslinkable hydrogels: emerging strategies for tissue engineering. Biomaterials 33 (5), 1281–1290. 10.1016/j.biomaterials.2011.10.067 22118821

[B212] Thakur A. Jaiswal M. K. Peak C. W. Carrow J. K. Gentry J. Dolatshahi-Pirouz A. (2016). Injectable shear-thinning nanoengineered hydrogels for stem cell delivery. Nanoscale 8 (24), 12362–12372. 10.1039/c6nr02299e 27270567

[B213] Thambi T. Li Y. Lee D. S. (2017). Injectable hydrogels for sustained release of therapeutic agents. J. Control. Release 267, 57–66. 10.1016/j.jconrel.2017.08.006 28827094

[B214] Thielen N. G. M. van der Kraan P. M. van Caam A. P. M. (2019). TGFβ/BMP signaling pathway in cartilage homeostasis. Cells 8 (9), 969. 10.3390/cells8090969 PMC6769927 31450621

[B215] Thomas J. Chopra V. Sharma A. Panwar V. Kaushik S. Rajput S. (2021). An injectable hydrogel having proteoglycan-like hierarchical structure supports chondrocytes delivery and chondrogenesis. Int. J. Biol. Macromol. 190, 474–486. 10.1016/j.ijbiomac.2021.08.226 34508717

[B216] Toh W. S. Lee E. H. Cao T. (2011). Potential of human embryonic stem cells in cartilage tissue engineering and regenerative medicine. Stem Cell Rev. Rep. 7 (3), 544–559. 10.1007/s12015-010-9222-6 21188652

[B217] Tonda-Turo C. Gnavi S. Ruini F. Gambarotta G. Gioffredi E. Chiono V. (2017). Development and characterization of novel agar and gelatin injectable hydrogel as filler for peripheral nerve guidance channels. J. Tissue Eng. Regen. Med. 11 (1), 197–208. 10.1002/term.1902 24737714

[B218] Torres-Figueroa A. V. Pérez-Martínez C. J. Encinas J. C. Burruel-Ibarra S. Silvas-Garcia M. I. Garcia Alegria A. M. (2021). Thermosensitive bioadhesive hydrogels based on poly(N-isopropylacrilamide) and poly(methyl vinyl ether-alt-maleic anhydride) for the controlled release of metronidazole in the vaginal environment. Pharmaceutics 13 (8), 1284. 10.3390/pharmaceutics13081284 34452245 PMC8402040

[B219] Tsumaki N. Okada M. Yamashita A. (2015). iPS cell technologies and cartilage regeneration. Bone 70, 48–54. 10.1016/j.bone.2014.07.011 25026496

[B220] Tu Y. Chen N. Li C. Liu H. Zhu R. Chen S. (2019). Advances in injectable self-healing biomedical hydrogels. Acta Biomater. 90, 1–20. 10.1016/j.actbio.2019.03.057 30951899

[B221] Tuan R. S. Chen A. F. Klatt B. A. (2013). Cartilage regeneration. J. Am. Acad. Orthop. Surg. 21 (5), 303–311. 10.5435/JAAOS-21-05-303 23637149 PMC4886741

[B222] Vayas R. Reyes R. Arnau M. R. Evora C. Delgado A. (2021). Injectable scaffold for bone marrow stem cells and bone morphogenetic protein-2 to repair cartilage. Cartilage 12 (3), 293–306. 10.1177/1947603519841682 30971092 PMC8236655

[B223] Vega S. L. Kwon M. Y. Burdick J. A. (2017). Recent advances in hydrogels for cartilage tissue engineering. Eur. Cell. Mat. 33, 59–75. 10.22203/eCM.v033a05 PMC5748291 28138955

[B224] Wang C. Wang Y. Wang C. Liu C. Li W. Hu S. (2021). Therapeutic application of 3B-PEG injectable hydrogel/Nell-1 composite system to temporomandibular joint osteoarthritis. Biomed. Mat. 17 (1), 015004. 10.1088/1748-605X/ac367f 34736242

[B225] Wang F. Chen J. Liu J. Zeng H. (2021). Cancer theranostic platforms based on injectable polymer hydrogels. Biomater. Sci. 9 (10), 3543–3575. 10.1039/d0bm02149k 33634800

[B226] Wang G. An Y. Zhang X. Ding P. Bi H. Zhao Z. (2021). Chondrocyte spheroids laden in GelMA/HAMA hybrid hydrogel for tissue-engineered cartilage with enhanced proliferation, better phenotype maintenance, and natural morphological structure. Gels 7 (4), 247. 10.3390/gels7040247 34940307 PMC8701895

[B227] Wang J. Li B. Pu X. Wang X. Cooper R. C. Gui Q. (2020). Injectable multicomponent biomimetic gel composed of inter-crosslinked dendrimeric and mesoporous silica nanoparticles exhibits highly tunable elasticity and dual drug release capacity. ACS Appl. Mat. Interfaces 12 (9), 10202–10210. 10.1021/acsami.0c01395 PMC10983814 32023033

[B228] Wang K-Y. Jin X-Y. Ma Y-H. Cai W. J. Xiao W. Y. Li Z. W. (2021). Injectable stress relaxation gelatin-based hydrogels with positive surface charge for adsorption of aggrecan and facile cartilage tissue regeneration. J. Nanobiotechnology 19 (1), 214. 10.1186/s12951-021-00950-0 34275471 PMC8287687

[B229] Wang Q. Li X. Wang P. Yao Y. Xu Y. Chen Y. (2020). Bionic composite hydrogel with a hybrid covalent/noncovalent network promoting phenotypic maintenance of hyaline cartilage. J. Mat. Chem. B 8 (20), 4402–4411. 10.1039/d0tb00253d 32242608

[B230] Wang Q. Wang Q. Teng W. (2016). Injectable, degradable, electroactive nanocomposite hydrogels containing conductive polymer nanoparticles for biomedical applications. Int. J. Nanomedicine 11, 131–144. 10.2147/IJN.S94777 26792990 PMC4708196

[B231] Wang Q-S. Xu B-X. Fan K-J. Fan Y. S. Teng H. Wang T. Y. (2021). Dexamethasone-loaded thermo-sensitive hydrogel attenuates osteoarthritis by protecting cartilage and providing effective pain relief. Ann. Transl. Med. 9 (14), 1120. 10.21037/atm-21-684 34430561 PMC8350682

[B232] Wang X. Wang Q. (2021). Enzyme-Laden bioactive hydrogel for biocatalytic monitoring and regulation. Acc. Chem. Res. 54 (5), 1274–1287. 10.1021/acs.accounts.0c00832 33570397

[B233] Wei M. Hsu Y-I. Asoh T-A. Sung M. H. Uyama H. (2022). Design of injectable poly(γ-glutamic acid)/chondroitin sulfate hydrogels with mineralization ability. ACS Appl. Bio Mat. 5 (4), 1508–1518. 10.1021/acsabm.1c01269 35286062

[B234] Wei P. Xu Y. Gu Y. Yao Q. Li J. Wang L. (2020). IGF-1-releasing PLGA nanoparticles modified 3D printed PCL scaffolds for cartilage tissue engineering. Drug Deliv. (Lond). 27 (1), 1106–1114. 10.1080/10717544.2020.1797239 PMC7470157 32715779

[B235] Wei W. Ma Y. Yao X. Zhou W. Wang X. Li C. (2021). Advanced hydrogels for the repair of cartilage defects and regeneration. Bioact. Mat. 6 (4), 998–1011. 10.1016/j.bioactmat.2020.09.030 PMC7557878 33102942

[B236] Wen C. Xu L. Xu X. Wang D. Liang Y. Duan L. (2021). Insulin-like growth factor-1 in articular cartilage repair for osteoarthritis treatment. Arthritis Res. Ther. 23 (1), 277. 10.1186/s13075-021-02662-0 34717735 PMC8556920

[B237] Werkmeister J. A. Adhikari R. White J. F. Tebb T. Le T. Taing H. (2010). Biodegradable and injectable cure-on-demand polyurethane scaffolds for regeneration of articular cartilage. Acta Biomater. 6 (9), 3471–3481. 10.1016/j.actbio.2010.02.040 20211278

[B238] Wong C. Y. Al-Salami H. Dass C. R. (2018). Microparticles, microcapsules and microspheres: A review of recent developments and prospects for oral delivery of insulin. Int. J. Pharm. X. 537 (1-2), 223–244. 10.1016/j.ijpharm.2017.12.036 29288095

[B239] Wu J. Chen Q. Deng C. Xu B. Zhang Z. Yang Y. (2020). Exquisite design of injectable hydrogels in cartilage repair. Theranostics 10 (21), 9843–9864. 10.7150/thno.46450 32863963 PMC7449920

[B240] Wu L. Cai X. Zhang S. Karperien M. Lin Y. (2013). Regeneration of articular cartilage by adipose tissue derived mesenchymal stem cells: Perspectives from stem cell biology and molecular medicine. J. Cell. Physiol. 228 (5), 938–944. 10.1002/jcp.24255 23042088

[B241] Xin H. (2022). Double-network tough hydrogels: A brief review on achievements and challenges. Gels 8 (4), 247. 10.3390/gels8040247 35448148 PMC9030633

[B242] Xu J. Liu Y. Hsu S-H. (2019). Hydrogels based on Schiff base linkages for biomedical applications. Molecules 24 (16), 3005. 10.3390/molecules24163005 PMC6720009 31430954

[B243] Xu L. Liu Y. Sun Y. Wang B. Xiong Y. Lin W. (2017). Tissue source determines the differentiation potentials of mesenchymal stem cells: A comparative study of human mesenchymal stem cells from bone marrow and adipose tissue. Stem Cell Res. Ther. 8 (1), 275. 10.1186/s13287-017-0716-x 29208029 PMC5718061

[B244] Xu X. Shi D. Liu Y. Yao Y. Dai J. Xu Z. (2017). *In vivo* repair of full-thickness cartilage defect with human iPSC-derived mesenchymal progenitor cells in a rabbit model. Exp. Ther. Med. 14 (1), 239–245. 10.3892/etm.2017.4474 28672920 PMC5488398

[B245] Xu Y. Xu Y. Bi B. Hou M. Yao L. Du Q. (2020). A moldable thermosensitive hydroxypropyl chitin hydrogel for 3D cartilage regeneration *in vitro* and *in vivo* . Acta Biomater. 108, 87–96. 10.1016/j.actbio.2020.03.039 32268237

[B246] Yan S. Wang T. Feng L. Zhu J. Zhang K. Chen X. (2014). Injectable *in situ* self-cross-linking hydrogels based on poly(L-glutamic acid) and alginate for cartilage tissue engineering. Biomacromolecules 15 (12), 4495–4508. 10.1021/bm501313t 25279766

[B247] Yan W. Xu X. Xu Q. Sun Z. Jiang Q. Shi D. (2020). Platelet-rich plasma combined with injectable hyaluronic acid hydrogel for porcine cartilage regeneration: A 6-month follow-up. Regen. Biomater. 7 (1), 77–90. 10.1093/rb/rbz039 32153994 PMC7053269

[B248] Yan X. Yang B. Chen Y. Song Y. Ye J. Pan Y. (2021). Anti-friction MSCs delivery system improves the therapy for severe osteoarthritis. Adv. Mat. 33 (52), e2104758. 10.1002/adma.202104758 34657320

[B249] Yang J. Jing X. Wang Z. Liu X. Zhu X. Lei T. (2021). *In vitro* and *in vivo* study on an injectable glycol chitosan/dibenzaldehyde-terminated polyethylene glycol hydrogel in repairing articular cartilage defects. Front. Bioeng. Biotechnol. 9, 607709. 10.3389/fbioe.2021.607709 33681156 PMC7928325

[B250] Yang J. Zhang Y. S. Yue K. Khademhosseini A. (2017). Cell-laden hydrogels for osteochondral and cartilage tissue engineering. Acta Biomater. 57, 1–25. 10.1016/j.actbio.2017.01.036 28088667 PMC5545789

[B251] Yao Y. Wang P. Li X. Xu Y. Lu G. Jiang Q. (2020). A di-self-crosslinking hyaluronan-based hydrogel combined with type I collagen to construct a biomimetic injectable cartilage-filling scaffold. Acta Biomater. 111, 197–207. 10.1016/j.actbio.2020.05.007 32434079

[B252] Yin S. Cao Y. (2021). Hydrogels for large-scale expansion of stem cells. Acta Biomater. 128, 1–20. 10.1016/j.actbio.2021.03.026 33746032

[B253] Yu J. Xu K. Chen X. Zhao X. Yang Y. Chu D. (2021). Highly stretchable, tough, resilient, and antifatigue hydrogels based on multiple hydrogen bonding interactions formed by phenylalanine derivatives. Biomacromolecules 22 (3), 1297–1304. 10.1021/acs.biomac.0c01788 33577294

[B254] Yu R. Zhang Y. Barboiu M. Maumus M. Noel D. Jorgensen C. (2020). Biobased pH-responsive and self-healing hydrogels prepared from O-carboxymethyl chitosan and a 3-dimensional dynamer as cartilage engineering scaffold. Carbohydr. Polym. 244, 116471. 10.1016/j.carbpol.2020.116471 32536386

[B255] Yu W. Hu B. Boakye-Yiadom K. O. Ho W. Chen Q. Xu X. (2021). Injectable hydrogel mediated delivery of gene-engineered adipose-derived stem cells for enhanced osteoarthritis treatment. Biomater. Sci. 9 (22), 7603–7616. 10.1039/d1bm01122g 34671794

[B256] Yu W. Zhu Y. Li H. He Y. (2020). Injectable quercetin-loaded hydrogel with cartilage-protection and immunomodulatory properties for articular cartilage repair. ACS Appl. Bio Mat. 3 (2), 761–771. 10.1021/acsabm.9b00673 35019280

[B257] Yuan F-Z. Wang H-F. Guan J. Fu J. N. Yang M. Zhang J. Y. (2021). Fabrication of injectable chitosan-chondroitin sulfate hydrogel embedding kartogenin-loaded microspheres as an ultrasound-triggered drug delivery system for cartilage tissue engineering. Pharmaceutics 13 (9), 1487. 10.3390/pharmaceutics13091487 34575563 PMC8472453

[B258] Yuan T. Li Z. Zhang Y. Shen K. Zhang X. Xie R. (2021). Injectable ultrasonication-induced silk fibroin hydrogel for cartilage repair and regeneration. Tissue Eng. Part A 27 (17-18), 1213–1224. 10.1089/ten.TEA.2020.0323 33353462

[B259] Yue K. Trujillo-de Santiago G. Alvarez M. M. Tamayol A. Annabi N. Khademhosseini A. (2015). Synthesis, properties, and biomedical applications of gelatin methacryloyl (GelMA) hydrogels. Biomaterials 73, 254–271. 10.1016/j.biomaterials.2015.08.045 26414409 PMC4610009

[B260] Zaviskova K. Tukmachev D. Dubisova J. Vackova I. Hejcl A. Bystronova J. (2018). Injectable hydroxyphenyl derivative of hyaluronic acid hydrogel modified with RGD as scaffold for spinal cord injury repair. J. Biomed. Mat. Res. A 106 (4), 1129–1140. 10.1002/jbm.a.36311 29266693

[B261] Zazakowny K. Lewandowska-Łańcucka J. Mastalska-Popławska J. Kaminski K. Kusior A. Radecka M. (2016). Biopolymeric hydrogels - Nanostructured TiO2 hybrid materials as potential injectable scaffolds for bone regeneration. Colloids Surf. B Biointerfaces 148, 607–614. 10.1016/j.colsurfb.2016.09.031 27694050

[B262] Zewail M. Nafee N. Helmy M. W. Boraie N. (2021). Synergistic and receptor-mediated targeting of arthritic joints via intra-articular injectable smart hydrogels containing leflunomide-loaded lipid nanocarriers. Drug Deliv. Transl. Res. 11 (6), 2496–2519. 10.1007/s13346-021-00992-9 34013458

[B263] Zhang F-X. Liu P. Ding W. Meng Q. B. Su D. H. Zhang Q. C. (2021). Injectable Mussel-Inspired highly adhesive hydrogel with exosomes for endogenous cell recruitment and cartilage defect regeneration. Biomaterials 278, 121169. 10.1016/j.biomaterials.2021.121169 34626937

[B264] Zhang M. Shi J. Xie M. Wen J. Niibe K. Zhang X. (2020). Recapitulation of cartilage/bone formation using iPSCs via biomimetic 3D rotary culture approach for developmental engineering. Biomaterials 260, 120334. 10.1016/j.biomaterials.2020.120334 32862124

[B265] Zhang Q. Xu H. Wu C. Shang Y. Wu Q. Wei Q. (2021). Tissue fluid triggered enzyme polymerization for ultrafast gelation and cartilage repair. Angew. Chem. Int. Ed. 60 (36), 19982–19987. 10.1002/anie.202107789 34173310

[B266] Zhang S. Huang D. Lin H. Xiao Y. Zhang X. (2020). Cellulose nanocrystal reinforced collagen-based nanocomposite hydrogel with self-healing and stress-relaxation properties for cell delivery. Biomacromolecules 21 (6), 2400–2408. 10.1021/acs.biomac.0c00345 32343129

[B267] Zhang T. Chen S. Dou H. Liu Q. Shu G. Lin J. (2021). Novel glucosamine-loaded thermosensitive hydrogels based on poloxamers for osteoarthritis therapy by intra-articular injection. Mater. Sci. Eng. C 118, 111352. 10.1016/j.msec.2020.111352 33254972

[B268] Zhang T. Tian T. Zhou R. Li S. Ma W. Zhang Y. (2020). Design, fabrication and applications of tetrahedral DNA nanostructure-based multifunctional complexes in drug delivery and biomedical treatment. Nat. Protoc. 15 (8), 2728–2757. 10.1038/s41596-020-0355-z 32669637

[B269] Zhang Y. Cao Y. Zhao H. Zhang L. Ni T. Liu Y. (2020). An injectable BMSC-laden enzyme-catalyzed crosslinking collagen-hyaluronic acid hydrogel for cartilage repair and regeneration. J. Mat. Chem. B 8 (19), 4237–4244. 10.1039/d0tb00291g 32270838

[B270] Zhang Y. Liu J. Huang L. Wang Z. Wang L. (2015). Design and performance of a sericin-alginate interpenetrating network hydrogel for cell and drug delivery. Sci. Rep. 5, 12374. 10.1038/srep12374 26205586 PMC4513302

[B271] Zhang Z. Li L. Yang W. Cao Y. Shi Y. Li X. (2017). The effects of different doses of IGF-1 on cartilage and subchondral bone during the repair of full-thickness articular cartilage defects in rabbits. Osteoarthr. Cartil. 25 (2), 309–320. 10.1016/j.joca.2016.09.010 27662821

[B272] Zhang Z. Lin S. Yan Y. You X. Ye H. (2021). Enhanced efficacy of transforming growth factor-β1 loaded an injectable cross-linked thiolated chitosan and carboxymethyl cellulose-based hydrogels for cartilage tissue engineering. J. Biomaterials Sci. Polym. Ed. 32 (18), 2402–2422. 10.1080/09205063.2021.1971823 34428384

[B273] Zhao H. Liu M. Zhang Y. Yin J. Pei R. (2020). Nanocomposite hydrogels for tissue engineering applications. Nanoscale 12 (28), 14976–14995. 10.1039/d0nr03785k 32644089

[B274] Zhao Z. Fang R. Rong Q. Liu M. (2017). Bioinspired nanocomposite hydrogels with highly ordered structures. Adv. Mat. 29 (45), 1703045. 10.1002/adma.201703045 29059482

[B275] Zheng D. Chen T. Han L. Lv S. Yin J. Yang K. (2022). Synergetic integrations of bone marrow stem cells and transforming growth factor-β1 loaded chitosan nanoparticles blended silk fibroin injectable hydrogel to enhance repair and regeneration potential in articular cartilage tissue. Int. Wound J. 2022. 10.1111/iwj.13699 PMC9284642 35266304

[B276] Zhong J. Guo B. Xie J. Deng S. Fu N. Lin S. (2016). Crosstalk between adipose-derived stem cells and chondrocytes: When growth factors matter. Bone Res. 4, 15036. 10.1038/boneres.2015.36 26848404 PMC4738199

[B277] Zhou J. Li J. Du X. Xu B. (2017). Supramolecular biofunctional materials. Biomaterials 129, 1–27. 10.1016/j.biomaterials.2017.03.014 28319779 PMC5470592

[B278] Zhou S. Bei Z. Wei J. Yan X. Wen H. Cao Y. (2022). Mussel-inspired injectable chitosan hydrogel modified with catechol for cell adhesion and cartilage defect repair. J. Mat. Chem. B 10 (7), 1019–1030. 10.1039/d1tb02241e 34994756

[B279] Zhou W. Lin J. Zhao K. Jin K. He Q. Hu Y. (2019). Single-cell profiles and clinically useful properties of human mesenchymal stem cells of adipose and bone marrow origin. Am. J. Sports Med. 47 (7), 1722–1733. 10.1177/0363546519848678 31100005

[B280] Zhou Y. Zhang Y. Dai Z. Jiang F. Tian J. Zhang W. (2020). A super-stretchable, self-healing and injectable supramolecular hydrogel constructed by a host-guest crosslinker. Biomater. Sci. 8 (12), 3359–3369. 10.1039/d0bm00290a 32374313

[B281] Zhu J. Yang S. Qi Y. Gong Z. Zhang H. Liang K. (2022). Stem cell-homing hydrogel-based miR-29b-5p delivery promotes cartilage regeneration by suppressing senescence in an osteoarthritis rat model. Sci. Adv. 8 (13), eabk0011. 10.1126/sciadv.abk0011 35353555 PMC8967232

[B282] Zhu Y. Ye L. Cai X. Li Z. Fan Y. Yang F. (2022). Icariin-loaded hydrogel regulates bone marrow mesenchymal stem cell chondrogenic differentiation and promotes cartilage repair in osteoarthritis. Front. Bioeng. Biotechnol. 10, 755260. 10.3389/fbioe.2022.755260 35223781 PMC8864219

[B283] Zinatloo-Ajabshir S. Morassaei M. S. Amiri O. Salavati-Niasari M. Foong L. K. (2020). Nd2Sn2O7 nanostructures: Green synthesis and characterization using date palm extract, a potential electrochemical hydrogen storage material. Ceram. Int. 46 (11), 17186–17196. Part A). 10.1016/j.ceramint.2020.03.014

[B284] Zinatloo-Ajabshir S. Mortazavi-Derazkola S. NdO-SiO nanocomposites (2018). Nd2O3-SiO2 nanocomposites: A simple sonochemical preparation, characterization and photocatalytic activity. Ultrason. Sonochem. 42, 171–182. 10.1016/j.ultsonch.2017.11.026 29429658

[B285] Zinatloo-Ajabshir S. Salavati-Niasari M. (2019). Preparation of magnetically retrievable CoFe2O4@SiO2@Dy2Ce2O7 nanocomposites as novel photocatalyst for highly efficient degradation of organic contaminants. Compos. Part B Eng. 174, 106930. 10.1016/j.compositesb.2019.106930

[B286] Zoratto N. Matricardi P. (2018). Semi-IPN- and IPN-based hydrogels. Adv. Exp. Med. Biol. 1059, 155–188. 10.1007/978-3-319-76735-2_7 29736573

